# The effect of AI-enabled virtual patient simulation on training outcomes and insecurities in psychotherapy education

**DOI:** 10.1186/s12909-026-09917-x

**Published:** 2026-07-15

**Authors:** Julia Cecil, Eesha Kokje, Anne-Kathrin Kleine, Insa Schaffernak, Lea Vogel, Selina Angerer, Eva Lermer, Susanne Gaube

**Affiliations:** 1 LMU Center for Leadership and People Management, Department of Psychology, LMU Munich, Munich, Germany; 2 Department of Business Psychology, Technical University of Applied Sciences Augsburg, Augsburg, Germany; 3 Department of Psychology, LMU Munich, Munich, Germany; 4 German Center for Mental Health (DZPG), partner site, Munich, Germany; 5 UCL Global Business School for Health, University College London, London, UK

**Keywords:** Artificial intelligence, Virtual patients, Psychotherapy training, Clinical skills

## Abstract

**Background:**

Preparing psychotherapy trainees for clinical complexity is a persistent educational challenge. Virtual patient (VP) simulations enabled by artificial intelligence (AI) may enhance training by offering practical, hands-on experience. This study examined their effectiveness in improving training outcomes and reducing insecurity in future psychotherapists.

**Methods:**

Psychology students and psychotherapy trainees (*N* = 87) were randomly assigned to four online 2D-VP sessions with automated feedback or pre-recorded role-play videos. Pre- and post-assessments measured perceived psychotherapeutic competence, self-efficacy, knowledge, and insecurity.

**Results:**

VP training significantly improved competence and self-efficacy while reducing insecurity, but did not enhance knowledge, though effects did not surpass video training. Greater clinical experience predicted higher competence and lower insecurity, but not training-related gains. Insecurity decreased earlier with video than with VP training. Simulation-related factors did not affect training outcomes.

**Conclusion:**

Design recommendations emphasize pre-briefings and enhancing VP authenticity to use VP simulations as a scalable, engaging complementary method in psychotherapy training.

**Supplementary Information:**

The online version contains supplementary material available at 10.1186/s12909-026-09917-x.

## Introduction

The global demand for mental health services has intensified over the past decade, placing considerable pressure on healthcare systems to increase access to psychotherapy [1]. This rising demand for mental health care has not been matched by growth in the workforce, resulting in a shortfall in trained therapists [2]. This gap underscores a deep structural challenge: developing efficient and effective methods to train enough new psychotherapists capable of meeting increasing service demands, while maintaining high standards of clinical education and professional preparation. One way to address this challenge is by using technology to support and extend traditional training. While traditional training may include some exposure to clinical cases, it typically provides limited opportunities for active, hands-on diagnostic practice. In particular, these considerations become highly relevant, as a growing body of research highlights the challenges faced by psychology students and psychotherapy trainees as they transition from academic study to clinical practice [3, 4]. Their early clinical experiences are often marked by feelings of insecurity, inadequacy, and self-doubt [4, 5]. These experiences are often connected with physical, emotional, and cognitive distress, such as negative emotions and doubts about professional competence [6]. For instance, over a third of trainees reported moderate to low life satisfaction alongside about half of the trainees reporting moderate to high perceived stress [7]. Similarly, a review found that around 60% of psychologists experience moderate to high stress [8].

A key contributor to these negative experiences appears to be a perceived disconnect between theoretical knowledge and clinical applicability, especially when facing complex or unfamiliar clinical scenarios [4]. Training opportunities are viewed as the most important factor in determining psychotherapists’ confidence and capability [9]. While traditional training formats such as lectures and seminars provide the foundation of clinical knowledge, they may fall short in preparing future therapists for the ambiguity, emotional demands, and relational complexity inherent to clinical work [10, 11]. Early and successful contact with patients may be especially beneficial in building self-efficacy, confidence, fostering professional identity, and establishing the foundation for long-term resilience [12]. Early clinical exposure, and thus more experience, is not only beneficial for psychotherapists but also has been linked to better treatment outcomes [13] and reduced patient dropout rates [14, 15]. However, providing high-quality, supervised clinical exposure during psychotherapy training can be resource-intensive and logistically complex [16]. In response, simulation-based education, which involves learning through realistic and interactive scenarios, for instance through virtual patients (VP), has emerged as a promising complementary approach, offering structured, repeatable, and safe learning environments [17].

VP simulations are interactive, computer-based tools that mimic real clinical scenarios to help learners develop diagnostic, therapeutic, and communication skills in a controlled environment [18–20]. They are designed to process written or spoken input from learners, enabling them to assume the role of a healthcare provider [21]. VP simulations provide access to standardized clinical scenarios that are challenging to practice in real-world settings, due to limited resources or patient availability, or beneficial to experience prior to direct patient contact [22]. Crucially, they offer a low-risk environment in which learners can make mistakes, reflect on decisions, and develop competence without jeopardizing patient safety [18, 22].

In medical education, the use of VP simulations is well-established and has consistently demonstrated benefits for diagnostic reasoning, communication, social skills, and decision-making [18–20]. By contrast, VP are not yet commonly used in psychotherapy training, but they may offer particular value in helping trainees develop diagnostic and interpersonal skills, build clinical confidence, and manage feelings of insecurity [23, 24]. While the evidence base is still small, a growing number of studies have begun to explore VP applications for training in psychiatric interviewing, suicide risk assessment, and therapeutic communication [25–29]. Studies indicate that VP training can improve various outcomes, ranging from self-efficacy to diagnostic accuracy [30, 31]. For instance, VP training led to sustained gains in mental health nurses’ clinical confidence and communication skills [31, 32]. Similarly, VP interventions, even those using non-interactive video recordings, support the acquisition of psychiatric knowledge and clinical reasoning, especially when repeated over time [27]. Compared to more observational and traditional lectures or text-based cases, VP simulations have led to superior learning outcomes in psychopathology, clinical communication, and self-efficacy [24]. However, findings are mixed when VP training is compared to more active in-person formats like role-play. Some studies show VP simulations, especially those using virtual reality, can outperform role-play in areas such as empathic communication [32]. Others find comparable benefits, with no significant differences between VP and role-play training in terms of increasing trainees’ self-efficacy, and symptom recognition [33]. Similarly, VP simulations appear equally effective as face-to-face simulated patients, offering a practical alternative when in-person training is not feasible [34].

Recent advances in AI, particularly in natural language processing, have improved the authenticity, responsiveness, and pedagogical value of VP simulations [28, 35]. In mental healthcare, VPs enabled by Large Language Models (LLM) have gained increasing attention, offering new opportunities for training and skill development [36, 37]. By incorporating theoretically grounded cognitive models, such as frameworks based on Cognitive Behavioral Therapy or disorder-specific literature, the VP’s behavior can be designed to reflect clinically plausible thought patterns, emotional responses, and interaction styles [28]. LLM-generated patient responses have been shown to be realistic and align naturally with the flow of interviews [36]. Compared to human patients, AI-based VPs provide a highly structured and consistent environment that enables participants to engage efficiently in active learning tasks, including problem-solving exercises [36]. Research shows that AI-supported VP practice can effectively enhance key counseling competencies, such as empathetic understanding and guided discovery [38]. A key aspect in understanding these effects is the role of simulation-related factors that influence VP effectiveness.

Research has highlighted several factors influencing the engagement with and effectiveness of simulation-based learning. Key determinants include user satisfaction with the simulation experience [39], perceived usability [40], perceived presence and immersion [41], and the degree of technology acceptance [42, 43]. Satisfaction with the simulation plays a central role in promoting learner engagement and motivation, particularly when platforms are intuitive and allow for active, self-directed learning [40]. Likewise, good usability reduces cognitive load, enabling learners to focus more effectively on clinical content [44]. A strong sense of presence, the state when virtual objects are perceived as actual objects [45], has also been linked to improved learning satisfaction and perceived realism [46]. Technological features such as speech recognition and enhanced VP facial or bodily expressions are connected to perceived authenticity and presence in VP training [47]. Further, technology acceptance, which depends on perceived usefulness and ease of use [48], has emerged as a critical factor influencing both learners’ engagement and their intention to integrate VP simulations into ongoing practice [42, 49].

Leveraging the advantages of AI-enabled systems and building on prior research, the present study examines the effectiveness of AI-enabled VP simulations in psychotherapy training. Psychology students and psychotherapy trainees from Germany and the UK were randomly assigned to either (a) an experimental group, where they completed four AI-enabled VP initial psychotherapy consultations with automated feedback after each session, or (b) a control group, where they passively viewed pre-recorded role-play videos. We investigate whether AI-enabled, interactive VP simulation training leads to greater improvements in perceived (i.e., pre- and post-intervention self-report measures) (1) *psychotherapeutic competence*, (2) *clinical self-efficacy*, (3) *knowledge* about mental disorders and reductions in (4) *insecurity* compared to video-based training (Hypotheses 1–4). We also explore if and how participants’ *practical experience* moderates the training effects across the different outcome measures (RQ1). Further, the effects on insecurities depending on the different patient cases used in the simulation and across the time points were analyzed (RQ2). Lastly, we examine to what extent simulation variables, such as satisfaction with the simulation experience, perceived usability, perceived presence and immersion, and technology acceptance influence the VP training (RQ3).

## Methods

### Participants

Master’s students in clinical psychology and psychotherapy trainees were recruited through flyers, social media, university mailing lists, and psychotherapy training institutes in Germany and the UK. A minimum sample size of 40 participants per group was estimated using the *simr* package [50] for a power (1- β-error probability) of 0.80. Based on prior studies, we assumed small to moderate effect sizes (with β ≈ 0.02) and small to moderate correlations between repeated measures [51].

In total, 136 participants started the study; 46 did not complete it, two were excluded for failing both attention checks, and one was removed for inconsistencies in their demographic data, resulting in a final sample of *N* = 87 (dropout rate = 33.8%). Key participant information is presented in Table 1. Dropout analysis revealed no significant differences in demographic variables between dropouts and completers (see Table S17 in the Supplementary Information).

**Table 1:** Participant demographics

	VP training (*n* = 44)	Video training (*n* = 43)	Total (*N* = 87)
	*N* (%) or *M* (*SD*)	*N* (%) or *M* (*SD*)	*N *(%) or *M* (*SD*)
Age	26.68 (6.14)	27.63 (6.51)	27.15 (6.3)
Gender			
Female	39 (88.6%)	40 (93%)	79 (90.8%)
Male	5 (11.4%)	3 (7%)	8 (9%)
Profession			
Student (Master’s in Clinical Psychology)	27 (61.4%)	25 (58.1%)	52 (59.8%)
Semester	3.07 (2.02)	3 (1.68)	3.04 (1.85)
Psychotherapy Trainee	17 (38.6%)	18 (40.9%)	35 (40.2%)
Supervision hours	36.88 (78.37)	19.33 (43.68)	27.86 (62.64)
Patient hours	197.21 (279.29)	48.24 (125.83)	115.52 (218.92)
Practical clinical experience (in years)	0.91 (1.1)	0.87 (1.49)	0.89 (1.3)
Previous VP simulation experience			
Yes	2 (5.5%)	1 (2.3%)	3 (3.4%)
No	42 (95.5%)	42 (97.7%)	84 (96.6%)

VP training = experimental group; Video training = control group. Participant demographics are reported for the baseline assessment

### Materials

Data for the preregistered study were collected via Qualtrics (Qualtrics, Provo, UT, USA) between February and June 2025. The VP simulation training sessions were conducted on the MedVR education platform (MedVREducation, Division of National Board of Examiners (NBME); Philadelphia, Pennsylvania, USA) and the role-play videos were sourced from YouTube (YouTube LLC, San Bruno, CA, USA). Both training formats were matched for session duration (approximately 15–20 min) and clinical content, with each session focusing on one of four common mental disorders: depression, panic disorder, generalized anxiety disorder (GAD), and adjustment disorder. The VP cases were based on anonymized first screening reports from the outpatient clinic of the MUNIP (Münchner Universitäre Ausbildungsinstitut für psychologische Psychotherapie [engl: Munich University Training Institute for Psychological Psychotherapy]) and chosen to portray the most frequently encountered diagnoses [52]. Each case was reviewed by one licensed psychotherapist to confirm the initial diagnostic impression. Full case descriptions are provided in the Supplementary Information. The MedVR education platform was selected because it supports standardized yet interactive simulation of clinical consultations and allowed the development of clinically relevant cases based on the anonymized screening reports from the collaborating psychotherapy institute. The role-play videos from YouTube were selected as a comparable observational learning format. The 15–20 min duration per session was chosen to mirror the portion of a typical first consultation devoted to diagnostic assessment, as the remainder of the session is usually spent on organizational matters. First consultations were selected because they allow for a more extensive exploration of patient history and thus opportunity for clinical questioning, enabling participants to practice more freely.

### System design and session procedure

**Fig. 1:**
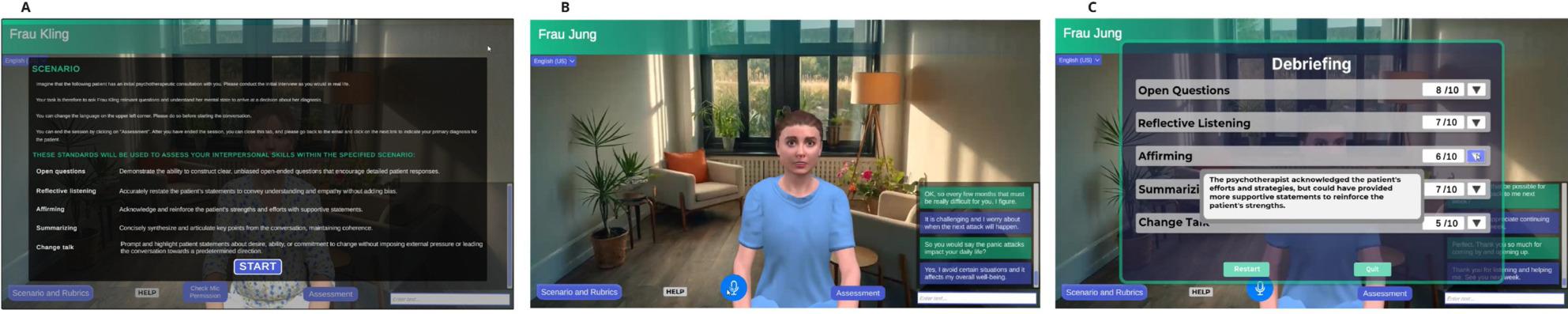
Elements of the VP Simulation interface. Note. **A** short prebriefing; **B** example of a real-time interaction with visible dialogue transcript; **C** automated feedback provided to participants. The screenshots are provided in full size in the Supplementary Information

For the VP simulation, participants first viewed a brief instructional video that explained how to navigate the platform and their task of interacting with the patient and arriving at an initial diagnosis (see Fig. 1A). Following this orientation, the VP interface was activated, and the simulated patient appeared on the screen. The simulated patient was a virtual agent that was displayed in 2D on the participants’ devices, comprising laptops and other computer-based devices, since the simulation environment was not supported on smartphones. The VP was sitting in a therapy room on a chair, facing the participant. Participants initiated the interaction by clicking on a button at the bottom of the screen. The simulation was in a speech-based format: participants provided spoken input, and the VP generated corresponding spoken responses. In parallel, a real-time transcript of the dialogue was displayed in the lower-right corner of the interface (see Fig. 1B). The VP system was based on a generative, transformer-based Large Language Model (LLM) trained by MedVREducation (Division of National Board of Examiners (NBME); Philadelphia, Pennsylvania, USA; see Fig. 1C). To leverage the advantages of immediate and personalized feedback through AI systems [53], participants received feedback following each session on five core Motivational Interviewing (MI) techniques [54]. In the following, “feedback” refers to this automated MI-based feedback. These techniques represent fundamental interpersonal and communication skills that are essential across a wide range of psychotherapeutic contexts, particularly during initial diagnostic and rapport-building phases. The feedback component was included as part of the system’s existing functionality, and MI techniques were used to ensure a standardized and well-defined feedback structure. To generate this feedback, the LLM was prompted to assess participants’ performance in these five MI techniques based on a description (see Supplementary Information) and to assign an overall performance score for each technique on a 1–10 scale. This approach follows previous work employing MI principles to structure automated feedback systems (e.g [55]). To assess the appropriateness of the automated ratings, one licensed psychotherapist reviewed a subset of four sessions. The psychotherapist rated the transcripts regarding each MI category on a scale from 1 to 10. The professional psychotherapist ratings aligned closely with the automated ratings, differing no more than one point, with the psychotherapist occasionally being slightly stricter. An exemplary conversation transcript, with the respective automated feedback, can be found in the Supplementary Information.

### Research design and procedure

**Fig. 2:**
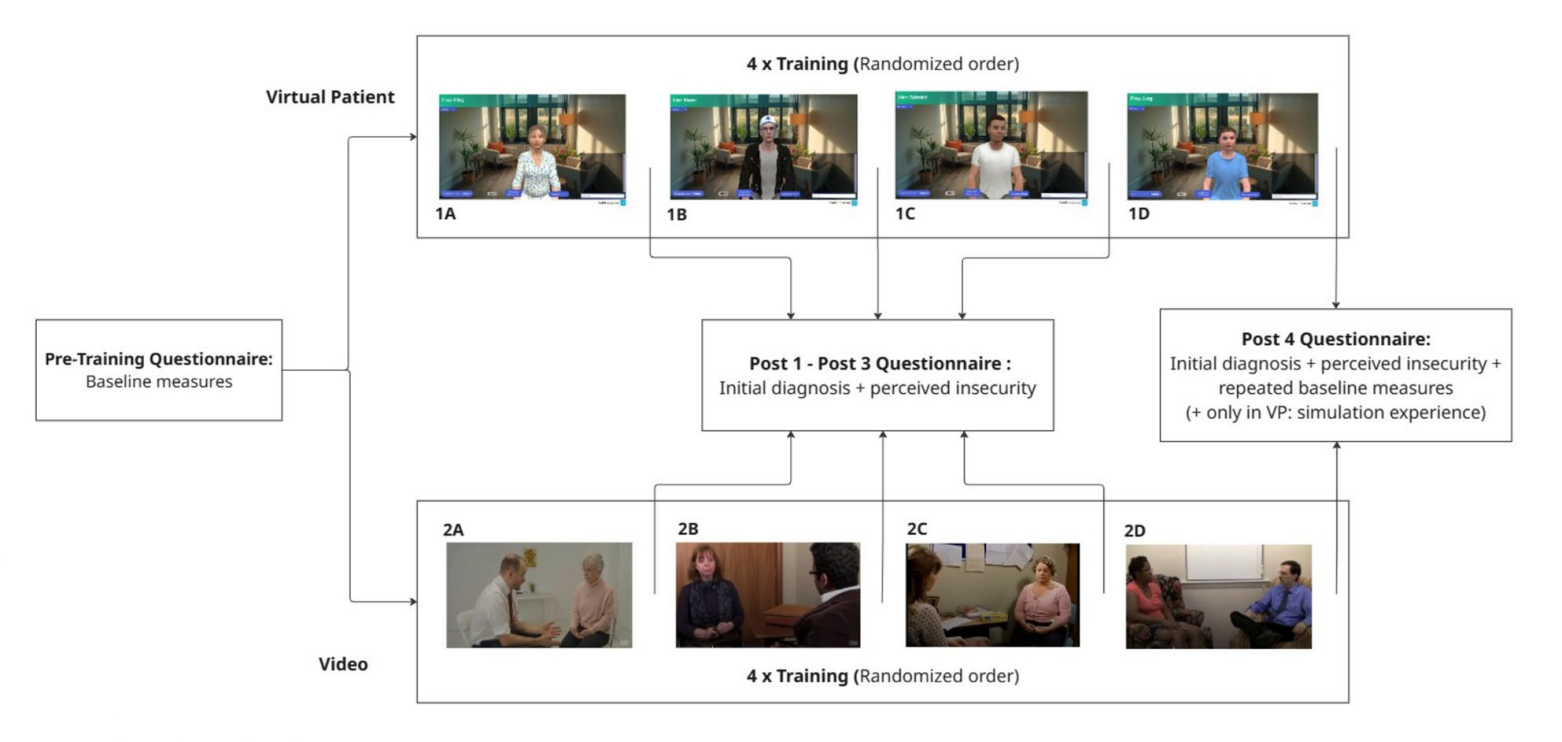
Overview of the study procedure. Notes. 1A – 1D: VP patients provided by MedVR Education (MedVREducation, Division of National Board of Examiners (NBME); Philadelphia, Pennsylvania, USA; 2A – 2D: Links to YouTube videos used as study materials: 2A: https://www.youtube.com/watch?v=8yFszPJClmw, 2B: https://www.youtube.com/watch?v=zpO_a463iOU, 2C: https://www.youtube.com/watch?v=4YhpWZCdiZc, 2D: https://www.youtube.com/watch?v=HIgQKH3Y1VE

Participants accessed the study through a registration link, where they received detailed information and provided informed consent. They were then randomly assigned to either the VP training or video training group, see Fig. 2. Sessions were spaced over multiple days to minimize participant burden and fatigue, reduce cognitive load, and allow time for learning consolidation. This approach aligns with research showing that practice is more effective when spaced out over time rather than massed in a single period [56]. Participants in the VP training group completed four interactive AI-enabled VP simulation training sessions on consecutive days, each featuring a different patient with a different mental disorder (patient case) with whom they held an initial psychotherapeutic conversation. In the video training group, participants watched four different psychotherapy role-play videos during the same timeframe. The videos depict standardized role-play interactions between two actors, portraying a psychotherapist and a patient, engaged in an initial psychotherapeutic session. These role-play videos were selected to enhance external validity, as standardized simulated interactions are commonly employed in contemporary psychotherapist training [57]. To control for potential order effects, the sequence of VP simulations or role-play videos was randomized for each participant. Standardized pacing was maintained by instructing participants to complete one session per day. Access to the session and post-session questionnaire for the following day was only granted once the respective post-session questionnaire was fully completed.

All participants completed a baseline (pre-training) questionnaire assessing key outcome variables described in the measures below. Each training session was followed by a brief post-session questionnaire (initial diagnosis and perceived insecurity as described in the measures below). After the fourth session, participants completed a final post-training questionnaire which repeated the baseline measures and, in the VP training group, assessed the user experience of the VP simulations with quantitative measures. Both groups subsequently could provide open-ended evaluations of the simulations. The questionnaires and video training sessions were conducted in English, and the VP simulation language could be adjusted from English to German. 

### Measures

The following variables were assessed at both baseline and post-intervention sessions. In addition, only perceived insecurity was measured after each training session to keep the sessions as brief as possible and to reduce participant burden and potential dropout. Simulation-related variables were assessed only following the final VP session.

#### Intervention measures

##### Perceived therapeutic competence

was measured using the 33-item Counseling Self-Estimate Inventory [58] (COSE), which uses a 6-point Likert scale ranging from 1 (“strongly disagree”) to 6 (“strongly agree”). Four subscales were included in the questionnaire: *microskills*, *counselling process integration*, *dealing with difficult client behavior*, and *awareness of values*. The fifth subscale, *cultural competence*, was omitted as the study context did not involve interactions with VP from diverse cultural, ethnic, or socioeconomic backgrounds. The instrument demonstrated an acceptable to high internal consistency (α = 0.66 − 0.92). Only the subscale *awareness of values* showed marginal sufficient internal consistency at pre-intervention (α = 0.53) and insufficient consistency at post-intervention (α = 0.40). Cronbach’s α is sensitive to the number of items, and short scales, as in our study with three to four items, often produce lower α values [59]. It has also been noted that α values below 0.50 can still be informative for theoretically grounded, validated scales [60]. As the present subscale is part of a widely validated instrument with demonstrated psychometric quality [58], we retained the subscale for further analysis, interpreting results cautiously. For each subscale and the overall score, mean values were calculated.

##### Perceived self-efficacy 

was measured using the Self-Efficacy Scale SE-12 [61], which consists of 12 items rated on a 10-point Likert scale from 1 (“very uncertain”) to 6 (“very certain”). This scale demonstrated high internal consistency (α = 0.89 − 0.90). For the overall score, mean values were calculated.

##### Knowledge of mental disorders 

was evaluated using multiple-choice questions based on the criteria of the International Statistical Classification of Diseases and Related Health Problems (10th revision, ICD-10 [62]) for each patients’ mental disorder: depression, generalized anxiety disorder (GAD), panic disorder, and adjustment disorder. Each disorder consisted of three questions with five response options. Participants received one point for each correct answer, zero points for incorrect answers, and the total score was converted into a percentage from 0% to 100%.

##### Perceived insecurity 

was assessed using five subdimensions of perceived clinical insecurity, with items rated on a 7-point Likert-type scale (1 = “very uncertain,” 7 = “very certain”). The five subdimensions (*insecurity regarding therapeutic relationship*, *complex symptoms*, *practical skills*, *oversights*, and *harm/suicidality*) were derived from a pre-study with *N* = 157 psychotherapists in training, consisting of one item each and representing the five most frequently reported insecurities. For each subscale and the overall score, mean values were calculated.

##### Perceived VP simulation assessment satisfaction with simulation experience 

was measured with the Satisfaction with Simulation Experience (SSE) Scale [63], consisting of two subscales (*clinical reasoning* and *clinical learning*) and items rated on a 5-point Likert scale (1 = “strongly agree” to 5 = “strongly disagree”). The scale has a good internal consistency of α = 0.84–0.85. For each subscale and the overall score, mean values were calculated.

##### Perceived presence within the virtual environment 

was measured with the Presence Scale from Makransky et al. [41]. This 10-item measure assessed perceived *physical* and *social presence* within the simulation, rated on a 5-point Likert scale (1 = “strongly disagree” to 5 = “strongly agree”) and an internal consistency of α = 0.79–0.87. The fourth subscale *self-presence* was omitted, as it mainly addresses embodiment aspects, i.e., the extent to which participants experience their virtual body as integrated with their physical body in immersive virtual reality. As the present study used a non-immersive 2D virtual simulation without a virtual body representation, this construct was not applicable and therefore not assessed. For each subscale and the overall score, mean values were calculated.

##### Perceived system usability 

was measured using the System Usability Scale (SUS) [64], with 10 items on a 5-point Likert scale, ranging from 1 = “strongly disagree” to 5 = “strongly agree”. The scale showed an internal consistency of α = 0.79. SUS scores were calculated by using the standard scoring procedure, in which transformed item responses are summed and multiplied by 2.5 to yield a total score ranging from 0 to 100 [64].

##### Technology acceptance 

was measured using the Technology Acceptance Model (TAM) [48]: This 8-item instrument assessed *perceived usefulness* and *ease of use*, using a 7-point Likert scale from 1 = “strongly disagree” to 5 = “strongly agree. The instrument demonstrated an internal consistency of α = .88 − .94. For each subscale and the overall score, mean values were calculated. Further, participants provided demographic information, including age, gender, and place of residence. They also reported their academic background or current psychotherapy training status, along with the extent of their clinical experience in years and prior experience with VP simulations (“yes”/”no”).

### Data analysis

All analyses were performed using R version 4.4.0 (R Core Team, 2024). To test hypotheses H1–H4, a series of linear mixed-effects models (LMMs) was conducted for the following dependent variables using *lme4* [65]: perceived psychotherapeutic competence and its four subdimensions, clinical self-efficacy, knowledge about mental disorders, and insecurities and their five subdimensions. In each model, time (pre-intervention vs. post-intervention) and group (experimental vs. control) were included as fixed effects. To account for interindividual variability and the hierarchical structure of repeated-measures data, participant ID was included as the random effect. P-values were adjusted using the Holm correction to account for multiple testing. Post hoc pairwise contrasts were calculated for both timepoints (pre-intervention and post-intervention; *emmeans* [66]. To contextualize the interaction results, we assessed the design sensitivity of the LMMs and computed the minimal statistically detectable interaction effects (MES) following the procedure outlined by Lakens [67]. These MES values were derived from the critical t value and the standard error of the time x group interaction and were additionally standardized using each model’s residual variance. Across measures, the observed MES ranged from 0.16 to 3.88 and the standardized MES were around *d* = 0.60 (see Table S16). The analyses are presented as planned, noting that smaller interaction effects could fall below the study’s sensitivity; readers are therefore encouraged to interpret the effect size estimated alongside the *p*-values.

To address RQ1 concerning practical experience as a moderator, clinical experience was incorporated as a level-2 moderator into the models from H1–H4 that exhibited significant effects. Moderator effects were examined by including interaction terms between group, time and clinical experience.

For RQ2, we conducted an exploratory analysis to examine whether the impact of perceived insecurities varied across different patient cases or over the four simulation timepoints. To test differences between patient cases, we conducted an ANOVA comparing a LMM including patient case (1–4) as a fixed effect and participant ID as a random effect against a null model without the patient case predictor, assessing whether patient case accounted for additional variance. As this analysis deviated slightly from the preregistered analysis, the rationale for this modification is detailed in the Supplementary Information. To assess differences across the timepoints, we calculated a LMM with timepoint (post-intervention 1–4) as the fixed effect and participant ID as random effect.

To exploratorily examine the influence of simulation-related variables on training outcomes (RQ3), we conducted multiple linear regression analyses using change scores in psychotherapeutic competence and clinical self-efficacy as dependent variables. Change scores were calculated as the difference between post- and pre-intervention measurements. Predictor variables included system usability, satisfaction with the simulation experience, perceived presence within the virtual environment, and technology acceptance. In addition, we conducted a qualitative inductive content analysis of participants’ open-ended responses to capture nuanced user experiences with the virtual patient system, following previous approaches [38, 68].

Requirements for all LMM analyses and the multiple linear regression model (RQ3) were checked, with assumptions generally met. Prior to all LMM analyses, we additionally assessed potential baseline differences between the experimental and control groups. Group differences in pre-intervention scores for each dependent variable were tested using independent-samples t-tests for normally distributed data, and Mann-Whitney-U test for non-normally distributed data, with normality assessed via the Shapiro–Wilk test.

## Results

**Fig. 3:**
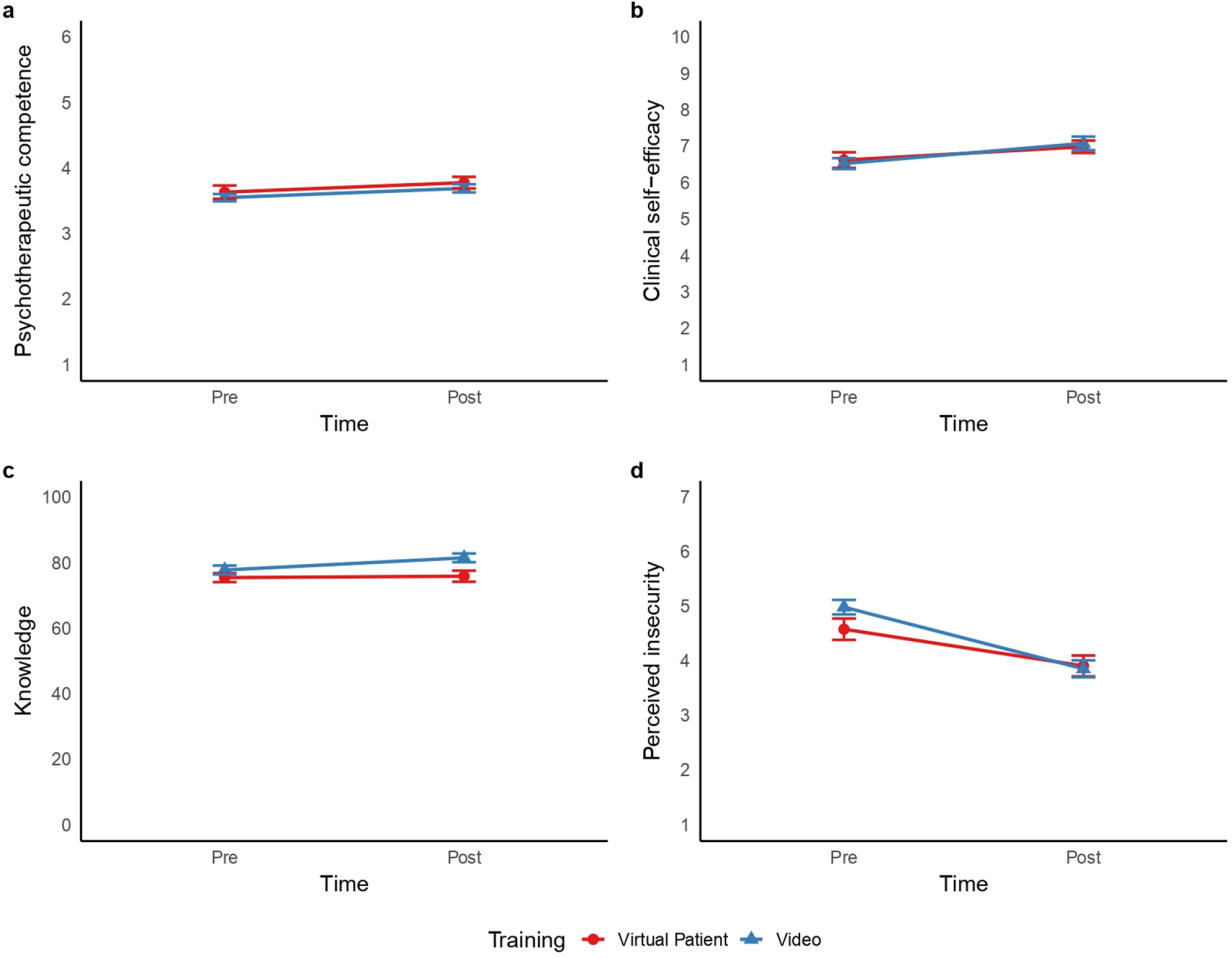
Mean main outcome values with standard errors at pre- and post-intervention. Notes. Figure **a** shows the psychotherapeutic competence, **b** the self-efficacy, **c** the participants’ knowledge about mental disorders, and **d** the perceived insecurity. The post-intervention measure was obtained after the completion of the fourth training session. Participants were instructed to complete the four training sessions across four consecutive days

As a preliminary step, we report descriptive statistics for all dependent variables, including the subscales, in Table S1 in the Supplementary Information. Figure 3 shows the mean and standard errors for the main dependent variables. Participants in both groups demonstrated moderate baseline levels of competence and clinical self-efficacy, which showed slight improvement following the training sessions. Baseline knowledge regarding mental disorders was relatively high; it showed a slight descriptive increase in the video training group, whereas it remained largely unchanged in the VP training group. Perceived insecurities were rated as moderate to high at baseline and decreased descriptively in both groups after the training interventions. Baseline comparisons between the VP training and video training groups revealed a significant difference only in overall insecurity (z = -2.12, *p* = .034), all other group differences were not significant (all *p* > .05), see Table S2 in the Supplementary Information.

### Comparable effects across both training groups

To account for the baseline difference of overall insecurity, we first computed LMMs using change scores (post-intervention minus pre-intervention scores) for each outcome variable, with group (VP vs. video training) as the sole fixed factor (see Table S3 in the Supplementary Information). These analyses yielded effect estimates comparable to our original LMMs with time (pre-intervention vs. post-intervention) and group as fixed effects described in the statistical analysis. Accordingly, we proceeded with the original LMMs for all subsequent analyses.

### Effects on perceived psychotherapeutic competence

A significant main effect of time was observed for *global psychotherapeutic competence* and the subscales *counseling process* and *dealing with difficult client behavior* (see Table 2). This indicates that participants across both conditions reported improvements in perceived competence after the intervention. However, no significant time × group interaction was found, indicating that, contrary to H1, the VP group did not show greater improvements in perceived competence. Including practical experience (mean-centered) in the regression model showed a positive main effect for *global competence*, indicating greater benefits for more experienced participants (see Table 3). However, no significant interaction was found, suggesting that experience did not moderate the training effects for perceived competence (RQ1). Within-group contrasts analyses showed significant improvements in the VP group for *global competence* (*estimate* = 0.15, *SE* = 0.06, *t*(83) = 2.70, *p* = .009), *counselling process* (*estimate* = 0.29, *SE* = 0.08, *t*(83) = 3.39, *p* = .001) and *dealing with difficult client behavior* (*estimate* = 0.24, *SE* = 0.08, *t*(83) = 3.13, *p* = .002). All within-group contrast analyses for the video training group can be seen in Table S4 in the Supplementary Information.

**Table 2:** Linear mixed effects model for psychotherapeutic competence and its subscales

Predictors		*β*	SE	t	*p*
Global competence					
	Intercept	3.55	0.08	44.22	< .001
	Group (VP)	0.08	0.11	0.75	1
	Time (Post)	0.14	0.06	2.56	.012
	Group x Time	0.00	0.08	0.03	1
Micro Skills					
	Intercept	3.87	0.11	36.12	< .001
	Group (EG)	0.01	0.15	0.06	1
	Time (Post)	-0.10	0.12	-0.15	1
	Group x Time	0.10	0.16	0.61	1
Counseling process					
	Intercept	3.28	0.12	27.89	< .001
	Group (EG)	0.14	0.17	0.86	1
	Time (Post)	0.16	0.09	1.82	.016
	Group x Time	0.12	0.12	1.02	1
Difficult client behavior					
	Intercept	2.87	0.10	27.90	< .001
	Group (EG)	0.10	0.14	0.70	1
	Time (Post)	0.28	0.08	3.53	< .001
	Group x Time	-0.04	0.11	-0.35	1
Awareness of values					
	Intercept	4.17	0.10	39.73	< .001
	Group (EG)	0.09	0.15	0.59	1
	Time (Post)	0.16	0.08	2.02	1
	Group x Time	-0.17	0.11	-1.59	1

Global Competence: Marginal R^2^ = 0.03; Conditional R^2^ = 0.77; Micro Skills: Marginal R^2^ = 0.00; Conditional R^2^ = 0.42; Counseling process: Marginal R^2^ = 0.04; Conditional R^2^ = 0.74; Difficult client behavior: Marginal R^2^ = 0.04; Conditional R^2^ = 0.73; Awareness of values: Marginal R^2^ = 0.00; Conditional R^2^ = 0.73

*p*-values were adjusted for multiple comparisons using the Holm correction

**Table 3:** Linear mixed effects model for psychotherapeutic competence and subscales with experience as a moderator

**Predictors **		***β***	**SE**	**t**	***p***
Global competence					
	Intercept	3.55	0.08	46.02	< .001
	Group (VP)	0.08	0.10	0.71	1
	Time (Post)	0.14	0.06	2.60	.002
	Experience	0.09	0.05	1.73	.034
	Group (VP) x Time (Post)	0.01	0.08	0.07	1
	Group (VP) x Experience	0.13	0.09	1.50	1
	Time (Post) x Experience	-0.05	0.04	-1.42	.460
	Group (VP) x Time (Post) x Experience	-0.02	0.06	-0.32	1
Micro skills					
	Intercept	3.87	0.11	36.30	< .001
	Group (EG)	0.00	0.15	0.03	1
	Time (Post)	-0.02	0.12	-0.16	.705
	Experience	0.11	0.07	1.50	.186
	Group (EG) x Time (Post)	0.10	0.16	0.60	1
	Group (EG) x Experience	-0.02	0.12	-0.18	1
	Time (Post) x Experience	-0.03	0.08	-0.41	1
	Group (EG) x Time (Post) x Experience	0.07	0.13	0.54	1
Counseling process					
	Intercept	3.28	0.12	28.42	< .001
	Group (EG)	0.13	0.16	0.83	1
	Time (Post)	0.16	0.08	1.84	.002
	Experience	0.04	0.08	0.51	.186
	Group (EG) x Time (Post)	0.13	0.12	1.08	1
	Group (EG) x Experience	0.26	0.13	2.02	1
	Time (Post) x Experience	-0.03	0.06	-0.51	.460
	Group (EG) x Time (Post) x Experience	-0.14	0.10	-1.46	1
Difficult client behavior					
	Intercept	2.88	0.10	28.88	< .001
	Group (EG)	0.10	0.14	0.66	1
	Time (Post)	0.28	0.08	3.55	< .001
	Experience	0.07	0.07	0.97	.062
	Group (EG) x Time (Post)	-0.04	0.11	-0.33	1
	Group (EG) x Experience	0.23	0.11	2.06	1
	Time (Post) x Experience	-0.02	0.05	-0.29	.535
	Group (EG) x Time (Post) x Experience	-0.12	0.09	-1.29	1

Global Competence: Marginal R^2^ = 0.12; Conditional R^2^ = 0.78; Micro Skills: Marginal R^2^ = 0.03; Conditional R^2^ = 0.4^2^; Counseling process: Marginal R^2^ = 0.09; Conditional R^2^ = 0.76; Difficult client behavior: Marginal R^2^ = 0.1^2^; Conditional R2 = 0.73; *p-*values were adjusted for multiple comparisons using the Holm correction. Given the non-significant results in the linear mixed effect model for awareness of values, no moderator analysis for professional experience was conducted

### Effects on perceived clinical self-efficacy

A significant main effect of time indicated increased *clinical self-efficacy* post-intervention (see Table 4). The non-significant time × group interaction did not support H2, which predicted greater self-efficacy improvements in the VP group compared to video-based training. Experience showed no moderating effect (Table 5), indicating that training benefits regarding self-efficacy did not differ based on participants’ practical experience (RQ1). Within-group contrasts showed significant pre-post increases in the VP group, estimate = 0.37, *SE* = 0.15, *t*(83) = 2.56, *p* = .012. Given these non-significant results, no moderator analyses for professional experience or within-group contrast analysis in the VP group were conducted.

**Table 4:** Linear mixed effects model for self-efficacy

**Predictors**		***β***	**SE**	**t**	***p***
Clinical self-efficacy					
	Intercept	6.53	0.18	35.67	< .001
	Group (VP)	0.10	0.26	0.37	1
	Time (Post)	0.56	0.15	3.74	.001
	Group x Time	-0.19	0.21	-0.91	1

Marginal R^2^ = 0.04; Conditional R^2^ = 0.68

*p*-values were adjusted for multiple comparisons using the Holm correction

**Table 5:** Linear mixed effects model for self-efficacy with experience as moderator

**Predictor**			***β***		**SE**	**t**	***p***
Clinical self-efficacy	Intercept		6.53		0.18	36.47	< .001
	Group (VP)		0.08		0.25	0.33	1
	Time (Post)		0.50		0.15	3.79	< .001
	Experience		0.06		0.12	0.52	.140
	Group (VP) x Time (Post)		-0.18		0.21	-0.89	1
	Group (VP) x Experience		0.44		0.20	2.15	1
	Time (Post) x Experience		-0.02		0.10	-0.17	.460
	Group (VP) x Time (Post) x Experience		-0.26		0.17	-1.59	1

Marginal R^2^ = 0.10; Conditional R^2^ = 0.70

*p*-values were adjusted for multiple comparisons using the Holm correction

### Effects on knowledge about mental disorders

No significant main or interaction effects emerged for knowledge about mental disorders (Table 6), failing to support H3, which predicted greater knowledge improvements in the VP group compared to video-based training. Given these non-significant results, no moderator analyses for professional experience or within-group contrast analysis in the VP group were conducted.

**Table 6:** Linear mixed effects model for knowledge about mental illnesses

**Predictors**		***β***	**SE**	**t**	***p***
Knowledge					
	Intercept	77.77	1.45	53.49	< .001
	Group (VP)	-2.32	2.04	-1.14	.894
	Time (Post)	3.72	1.39	2.68	1
	Group x Time	-3.29	1.95	-1.69	1

Marginal R^2^ = 0.03; Conditional R^2^ = 0.77

*p*-values were adjusted for multiple comparisons using the Holm correction

### Effects on perceived insecurities

A significant main effect of time emerged for *overall insecurity* and all subdomains except *insecurity in building a therapist-patient relationship* (see Table 7). The non-significant time × group interaction did not support H4, which predicted greater insecurity reductions in the VP group. Including professional experience showed a negative main effect on *overall insecurity* and *insecurity regarding practical skills*, with more experienced participants reporting lower insecurities. However, no significant interaction effects emerged, indicating experience did not moderate training benefits on reducing insecurities (RQ1, Table 8). Within-group contrasts showed significant pre-post reductions in the VP group for *overall insecurity* (estimat*e* = − 0.68, *SE* = 0.19, *t*(83) = − 3.66, *p* < .001), *insecurity in dealing with complex symptoms* (estimate = − 1.03, *SE* = 0.25, *t*(83) = − 4.07, *p* < .001), *insecurity about practical skills* (estimate = − 1.06, *SE* = 0.23, *t*(83) = − 4.70, *p* < .001), *insecurity about oversights* (estimate = − 0.55, *SE* = 0.24, *t*(83) = − 2.23, *p* = .028), and *insecurity regarding harm/ suicidality* (estimate = − 0.78, *SE* = 0.26, *t*(83) = − 3.03, *p* = .003).

**Table 7:** Linear mixed effects model for insecurity and subscales

**Predictors**			***β***		**SE**	**t**	***p***
Overall insecurities							
	Intercept		4.99		0.17	28.90	< 0.001
	Group (VP)		-0.40		0.24	-1.67	1
	Time (Post)		-1.13		0.19	-5.93	< 0.001
	Group x Time		0.46		0.27	1.70	1
Building relationship							
	Intercept		4.21		0.24	17.47	< 0.001
	Group (EG)		-0.48		0.34	-1.42	1
	Time (Post)		-0.79		0.28	-2.87	1
	Group x Time		0.81		0.39	2.08	1
Complex symptoms							
	Intercept		5.42		0.28	23.86	< 0.001
	Group (EG)		-0.28		0.32	-0.88	1
	Time (Post)		-1.40		0.25	-5.48	< 0.001
	Group x Time		0.37		0.36	1.04	1
Practical skills							
	Intercept		5.61		0.19	28.89	< 0.001
	Group (EG)		-0.45		0.27	-1.63	1
	Time (Post)		-1.42		0.23	-6.12	< 0.001
	Group x Time		0.37		0.33	1.15	1
Oversights							
	Intercept		5.26		0.20	26.10	< 0.001
	Group (EG)		-0.39		0.28	-1.39	1
	Time (Post)		-1.09		0.24	-4.48	< 0.001
	Group x Time		0.55		0.34	1.59	1
Harm/ Suicidality							
	Intercept		4.44		0.25	17.62	< 0.001
	Group (EG)		-0.42		0.35	-1.18	1
	Time (Post)		-0.95		0.27	-3.52	0.001
	Group x Time		0.18		0.38	0.47	1

Overall insecurity: Marginal R^2^ = 0.15; Conditional R^2^ = 0.48; Building relationship: Marginal R^2^ = 0.04; Conditional R^2^ = 0.37; Complex symptoms: Marginal R^2^ = 0.15; Conditional R^2^ = 0.46; Practical skills: Marginal R^2^ = 0.20; Conditional R^2^ = 0.43; Oversights: Marginal R^2^ = 0.10; Conditional R^2^ = 0.33; Harm/Suicidality: Marginal R^2^ = 0.07; Conditional R^2^ = 0.47

*p*-values were adjusted for multiple comparisons using the Holm correction

**Table 8:** Linear mixed effects model for insecurity and subscales with experience as moderator

**Predictors**			***β***	**SE**	**t**	***p***
Overall insecurities	Intercept		4.98	0.17	30.04	< 0.001
	Group (VP)		-0.39	0.23	-1.67	1
	Time (Post)		-1.13	0.19	-6.00	< 0.001
	Experience		-0.18	0.11	-1.56	0.021
	Group (VP) x Time (Post)		0.45	0.26	1.70	1
	Group (VP) x Experience		-0.37	0.19	-1.96	1
	Time (Post) x Experience		0.12	0.13	0.90	0.460
	Group (VP) x Time (Post) x Experience		0.21	0.21	0.97	1
Building Relationship						
	Intercept		4.20	0.24	17.76	< 0.001
	Group (EG)		-0.47	0.33	-1.39	1
	Time (Post)		-0.79	0.27	-2.89	0.149
	Experience		-0.29	0.16	-1.82	0.122
	Group (EG) x Time (Post)		0.80	0.38	2.09	0.522
	Group (EG) x Experience		-0.24	0.27	-0.90	1
	Time (Post) x Experience		0.15	0.19	0.83	0.460
	Group (EG) x Time (Post) x Experience		0.28	0.31	0.91	1
Complex Symptoms						
	Intercept		5.42	0.22	24.33	< 0.001
	Group (EG)		-0.27	0.31	-0.86	1
	Time (Post)		-1.40	0.26	-5.47	< 0.001
	Experience		-0.10	0.15	-0.64	0.077
	Group (EG) x Time (Post)		0.37	0.36	1.03	1
	Group (EG) x Experience		-0.43	0.25	-1.71	1
	Time (Post) x Experience		-0.10	0.17	-0.60	1
	Group (EG) x Time (Post) x Experience		0.34	0.29	1.18	1
Practical skills						
	Intercept		5.60	0.18	30.40	< 0.001
	Group (EG)		-0.43	0.26	-1.65	1
	Time (Post)		-1.42	0.23	-6.23	< 0.001
	Experience		-0.16	0.13	-1.27	0.007
	Group (EG) x Time (Post)		0.36	0.32	1.13	1
	Group (EG) x Experience		-0.56	0.21	-2.67	0.330
	Time (Post) x Experience		0.10	0.15	0.65	0.373
	Group (EG) x Time (Post) x Experience		0.37	0.26	1.42	1
Oversights						
	Intercept		5.26	0.20	26.04	< 0.001
	Group (EG)		-0.39	0.28	-1.36	1
	Time (Post)		-1.09	0.25	-4.42	< 0.001
	Experience		-0.05	0.14	-0.36	0.357
	Group (EG) x Time (Post)		0.55	0.35	1.57	1
	Group (EG) x Experience		-0.18	0.23	-0.80	1
	Time (Post) x Experience		0.08	0.17	0.47	1
	Group (EG) x Time (Post) x Experience		-0.08	0.28	-0.30	1
Harm/ Suicidality						
	Intercept		4.44	0.24	18.13	< 0.001
	Group (EG)		-0.40	0.34	-1.16	1
	Time (Post)		-0.95	0.26	-3.61	< 0.001
	Experience		-0.28	0.17	-1.68	0.123
	Time (Post) x Time (Post)		0.16	0.37	0.44	1
	Group (EG) x Experience		-0.43	0.28	-1.57	1
	Time (Post) x Experience		0.35	0.18	1.96	0.085
	Group (EG) x Time (Post) x Experience		0.13	0.30	0.44	1

Overall insecurity: Marginal R^2^ = 0.23; Conditional R^2^ = 0.50; Building relationship: Marginal R^2^ = 0.09; Conditional R^2^ = 0.39; Complex symptoms: Marginal R^2^ = 0.19; Conditional R^2^ = 0.47; Practical skills: Marginal R^2^ = 0.29; Conditional R^2^ = 0.46; Oversights: Marginal R^2^ = 0.11; Conditional R^2^ = 0.34; Harm/Suicidality: Marginal R^2^ = 0.14; Conditional R^2^ = 0.51

*p*-values were adjusted for multiple comparisons using the Holm correction

Additionally, we ran further analyses to test the robustness of our findings. Given that patient hours are a component of the prior moderator practical experience (see Note in Table 1), we included them as a moderator to capture both their shared and potentially unique contribution. To account for the baseline differences observed in overall insecurity, we calculated the original LMMs controlled for baseline insecurity. Further, we controlled the models for study completion time. Including patient hours (see Tables S8-S10), initial insecurities (see Tables S11-S14), and study completion time (see Tables S18-21) did not change the patterns of the results. We were also interested in understanding how the automated feedback provided by the VP might have influenced the outcomes in the VP group. Accordingly, we compared the models with feedback with the models without feedback using a likelihood-ratio test (ANOVA). Including mean feedback led to a significant model improvement only for the subdimension awareness of values and no significant improvement for any other outcomes (see Table S15).

### Insecurity differences in patient cases and across time points

**Fig. 4:**
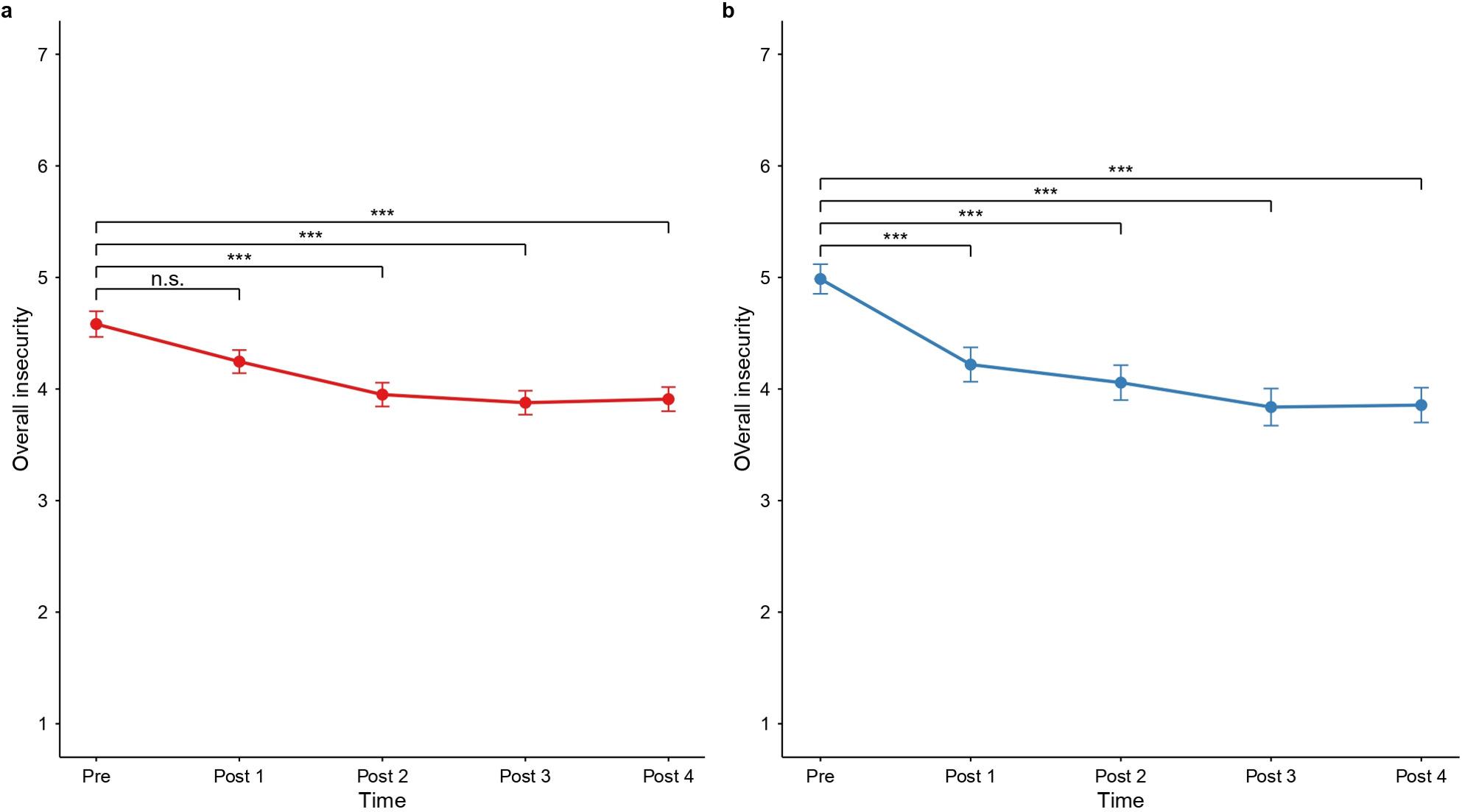
Mean values for insecurity across all time points for **a** VP training and **b** video training. Notes. Post-intervention assessments (Post 1–4) were conducted following each of the four training sessions, with Post 1 following the first session, Post 2 following the second, and so forth. Participants were instructed to complete the four sessions over four consecutive days.

We further explored exploratively whether the effects of perceived insecurities varied by patient case and across the four post-intervention timepoints (RQ2). Model comparisons with the null model indicated that including patient case as a fixed factor significantly improved model fit for the VP training group (𝜒^2^ (3) = 7.90, *p* = .048), but not for the video training group (𝜒^2^ (3) = 1.56, *p* = .667). This points to systematic variation in perceived insecurities across patient cases in the VP condition. Nevertheless, post-hoc pairwise comparisons of estimated marginal means (Holm-corrected) did not identify significant differences between any specific patient cases (all adjusted 𝑝 ≥ 0.05; see Table S5 & S6 in the Supplementary Information). However, descriptively, the patient case involving suicidal tendencies elicited the highest post-interaction insecurity levels. Across timepoints, the VP group showed significant insecurity reduction that persisted throughout only after the second session (Fig. 4; Table S7 in the Supplementary Information), indicating multiple VP interactions are necessary to achieve an effect. Video training showed significant reductions already after one session (Fig. 4; Table S7 in the Supplementary Information).

### The influence of simulation-related variables

Descriptive statistics (Table 9) showed moderate satisfaction with VP simulation, with clinical learning rated higher than clinical reasoning. Physical and social presence were low to moderate. System usability was rated as good, with participants finding the system useful and easy to use. To explore how simulation-related variables affect training gains (RQ3), we ran exploratory multiple linear regression for changes in the main outcome variables global psychotherapeutic competence, clinical self-efficacy, knowledge about mental illnesses, and perceived insecurity. The results showed that none of the simulation-related variables significantly predicted changes in these outcomes (Table 10). This indicates that perceptions of usability, satisfaction with the simulation, presence, and technology acceptance have little to no influence on these outcomes.

**Table 9:** Means and standard deviation of simulation variables

**Variable**	**Subscale**	***M*** **(*SD*)**
Satisfaction with simulation		3.83 (0.73)
	Clinical learning	4.06 (0.75)
	Clinical reasoning	3.60 (0.85)
Presence		2.54 (0.78)
	Physical presence	2.58 (0.77)
	Social presence	2.50 (0.85)
System Usability		77.05 (17.53)
Technology Acceptance		4.95 (1.03)
	Perceived usefulness	4.31 (1.47)
	Perceived ease of use	5.6 (1.02)

*N* = 44; Satisfaction with simulation measured on a 1–5 scale. Presence measured on a 1–5 scale. System Usability Scale (SUS) scored from 0–100. Technology Acceptance measured on a 1–7 scale; *M* = Mean; *SD* = Standard deviation

**Table 10:** Multiple linear regression analyses for changes in global competence and self-efficacy

	**Predictors**	***β***	**SE**	**t**		***p***
Global competence						
	Intercept	-0.31	0.41	-0.76		0.454
	Simulation satisfaction	0.13	0.14	0.93		1
	Presence	-0.17	0.09	-1.87		0.966
	System usability	0.00	0.00	0.75		1
	Technology acceptance	0.02	0.06	0.38		1
Self-efficacy						
	Intercept	-0.22	1.09	-0.20		1
	Simulation satisfaction	-0.04	0.36	-0.12		1
	Presence	-0.23	0.24	-0.97		1
	System usability	0.01	0.01	0.74		1
	Technology acceptance	0.13	0.17	0.75		1
Knowledge						
	Intercept	3.02	9.68	0.31		0.757
	Simulation satisfaction	4.38	3.19	1.37		1
	Presence	-0.69	2.14	-0.32		1
	System usability	-0.09	0.12	-0.74		1
	Technology acceptance	-2.16	1.51	-1.43		1
Overall Insecurity						
	Intercept	1.29	1.46	0.88		0.385
	Simulation satisfaction	0.30	0.48	0.62		1
	Presence	0.63	0.32	1.95		0.871
	System usability	-0.05	0.02	-2.54		0.246
	Technology acceptance	-0.23	0.23	-1.01		1

Global competence: R^2^ = 0.15; Adjusted R^2^ = 0.07; F(4, 38) = 1.76; *p* = .157; Self-efficacy: R^2^ = 0.06; Adjusted R^2^ = -0.04; F(4, 38) = 0.61; *p* = .657; Knowledge: R^2^ = 0.07; Adjusted R^2^ = -0.02; F(4, 38) = 0.76; *p* = .560; Insecurity: R^2^ = 0.26; Adjusted R^2^ = 0.18; F(4, 38) = 3.37; *p* = .019

*p*-values of the predictors were adjusted for multiple comparisons using the Holm correction

### Qualitative findings

Through inductive analysis of participants’ open-ended responses, we identified both positive and negative experiences that characterize interactions with the virtual patient system.

#### Conversational flow & realism

Participants frequently commented on the quality and realism of the system’s responses. Many described the dialogue as coherent and contextually appropriate, noting “very precise answers to the questions asked” (P4) and highlighting the value for practicing communication in a low-stakes environment. At the same time, several participants perceived limitations in conversational depth or human-likeness. Responses were sometimes described as “really short,” lacking detail or spontaneity, with one participant noting that “real patients would give longer answers, maybe also have questions” (P1). Some also mentioned repetitive or overly stereotypical phrasing, which reduced perceived authenticity.

#### Usability & interaction flow

The interface and overall interaction sequence were broadly experienced as intuitive and easy to navigate. Participants appreciated that “it was easy to use, only a few steps to follow” (P7). However, certain interaction elements created friction. For instance, unexpected navigation behaviors such as the session ending when pressing “assessment” (P12).

#### Technical performance

Fast response times and accurate speech recognition facilitated smooth conversations for many participants. Nevertheless, technical issues occasionally disrupted the interaction. These included delayed responses, misrecognitions, or missing replies, with one participant noting: “Sometimes words or phrases would show up differently in text form, probably due to speaking too quietly for the system to pick the words up correctly” (P5).

#### Social presence & avatar representation

Participants expressed negative sentiments regarding the virtual patient’s social presence. They viewed the avatar’s mimics and gestures as a limiting factor for a sense of natural interpersonal interaction. They described the avatar as lacking human-like expressivity, with one participant stating, “The patient looks a little bit creepy” (P7).

#### Engagement, learning value & case design

Many participants emphasized the novelty and motivational aspects of interacting with an AI-enabled VP. They highlighted the learning value, with one participant commenting that the system provided “a truly safe setting” (P36) to practice communication skills prior to real-world role-plays. The experience was described as engaging and potentially something they “would use more often if it was available” (P23). Participants also highlighted that the automated feedback was immediate and structured, allowing them to identify areas for improvement. Overall, the qualitative findings indicate that the VP system was perceived as an interesting and effective educational tool, while also revealing areas with potential for improvement.

## Discussion

This study compared VP simulation and observational video role-play training in improving psychotherapeutic capabilities, as well as in reducing perceived insecurities in psychotherapy trainees and master’s students in psychology. VP training enhanced competence and self-efficacy while reducing insecurity, but did not improve knowledge. However, the effects did not exceed more traditional, observational video training. The level of clinical experience was associated with higher competence and lower insecurity independent of training effects. The four different patient cases proved beneficial in reducing insecurity in both VP and video training conditions. Surprisingly, insecurity decreased earlier with video training. Simulation-related variables, such as presence and system usability did not influence the training effectiveness.

Interaction with the VP led to significant gains in most learning outcomes, aligning with previous evidence of the positive educational impact of VP training [24, 31, 32]. These gains likely stem from the interactive and applied nature of VP training, which supports clinical skill development [24]. Participants in our study described the VP simulations as engaging opportunities to apply their theoretical knowledge in practice within a safe environment, echoing prior findings [22]. However, participants did not consistently show improvements across all outcome measures; there were no significant gains in awareness of values, micro skills or knowledge acquisition, nor any significant reduction in perceived insecurity related to therapeutic relationships. The absence of significant changes in *value awareness* and *relationship-related insecurity* may be attributable to the nature of these constructs, which typically evolve over the course of therapy rather than being established after the first session [69]. Initial therapy sessions are typically structured to explore patients’ problems, goals, and diagnoses, with less emphasis on cultivating the therapeutic relationship or addressing the therapist’s values [70]. Participants in our study similarly reported focusing primarily on the diagnostic aspects during these sessions. Consequently, the emphasis on information gathering may have diverted attention from relational dynamics, thus limiting growth in these more relationally sensitive domains. The lack of improvement in *microskills* may follow a similar pattern: as participants were tasked with formulating a preliminary diagnosis for the VP, they focused on gathering information rather than formulating brief, precise questions. The VP system’s capability to interpret and respond even to imprecise or ambiguously phrased input likely further reduced the incentive to phrase questions carefully, as it was designed to provide an appropriate response to any participant input. Moreover, AI-enabled VP often display a positivity bias, tending toward agreeableness and compliance and thus, provide information even without explicit prompting [28]. Participants’ system evaluations echoed that the VP’s cooperative behavior reduced their need for precise question formulation. Further, the absence of gains in *knowledge about mental disorders* may be explained by the cognitive complexity of VP training. Participants were required to navigate the simulation interface, manage the diagnostic task, and engage in the conversation with the patient simultaneously. This multitasking likely imposed a high cognitive load, which is known to limit the capacity to process and consolidate new information [71]. Previous studies have also shown that VP simulations can be perceived as cognitively demanding, particularly by learners with limited prior knowledge [71]. Consistent with this, some participants’ system evaluations indicated that interacting with the VP was demanding and confusing, particularly because the interface displayed a live transcription of the conversation.

Importantly, VP training did not outperform video-based training. Participants in the video training group achieved comparable gains across all measured outcomes, supporting prior evidence that alternative formats, such as video simulations, can match or exceed VP simulations in certain contexts [34, 72]. Research shows that effective simulation-based learning depends not only on the technology itself but also on robust pedagogical framing, including pre-briefings, case-specific orientation, and structured debriefings [17, 73]. In our study, these elements were minimal: the pre-briefing was limited to a short written paragraph, and the available instructional video was generic and not tailored to the patient cases. Moreover, although participants received automated feedback from an LLM after each session, this feedback might have lacked the adaptive and dialogic qualities of human facilitation that are particularly important for complex communication skills [73].

In contrast, the video-based training provided a clear, well-structured model of communication without placing additional cognitive demands on learners to navigate technology or interpret automated feedback. From the perspective of Cognitive Load Theory (CLT) [74], these videos functioned as worked examples that likely reduced extraneous cognitive load and supported the acquisition of initial schemas for conducting initial therapy sessions. By presenting a transparent, step-by-step solution path, the videos might have directed participants’ attention toward understanding and internalizing the demonstrated procedures, thereby freeing cognitive resources for learning relevant to processes. In contrast, participants in the VP simulation condition were required to generate appropriate responses autonomously, which likely imposed higher cognitive load. Within the CLT framework, this pattern is consistent with the expertise reversal effect [18, 75], whereby worked examples are particularly effective for learners with limited prior knowledge, whereas advanced learners profit more from self-directed tasks [74]. As our participants were novices rather than licensed psychotherapists, they likely benefited more from worked examples (i.e., the videos), than from the VP simulation. Taken together, the limited pedagogical scaffolding, non-interactive feedback and cognitive load in the VP condition may have constrained its effectiveness, explaining why VP training did not produce greater gains than video-based training.

Greater clinical experience was linked to higher global competence, lower overall insecurity, and insecurity related to practical skills. However, the extent of clinical experience did not influence how much participants benefited from the training. This lack of differential effects indicates that all participants in this cohort - both psychology master’s students and the somewhat more experienced psychotherapy trainees - benefitted from the VP simulation, demonstrating its broad applicability. These findings align with research suggesting that VP simulations are particularly valuable for skill development in early training phases [17]. By grounding learning in realistic contexts, such approaches have the potential to promote both retention and practical application.

No significant differences in insecurity reduction emerged between patient case types across the VP and video groups. This suggests that, particularly in early training phases, the overall experience of engaging with a VP is sufficient to reduce insecurity, regardless of the specific clinical scenario. The four cases used in the simulation reflected the most common diagnoses [51], yet the case itself did not appear to drive the effect. Descriptive analyses, however, suggest that the patient case involving suicidal tendencies elicited the highest post-interaction insecurity levels. This aligns with previous findings showing that encounters with real patients exhibiting suicidal behavior can heighten emotional burden [76], and suggests that similar effects may occur when interacting with such VP. Nevertheless, given that post hoc contrasts did not reach statistical significance after Holm correction, this observation should be interpreted with caution.

Notably, timepoint comparisons revealed that insecurity in the VP training group decreased significantly only after the second session, whereas the video training group already showed improvements in the first session. This delayed effect in the VP condition may reflect an initial adjustment period required to adapt to the interactive and cognitively more demanding VP format [77]. In contrast, video training may have enabled faster insecurity reductions by allowing participants to focus directly on modeled behaviors. These findings again highlight the importance of instructional design in helping learners navigate simulation-based training. Early gains in the VP training group may be facilitated by incorporating structured pre-briefings to help acclimate to the VP format.

Participants’ quantitative evaluations of the VP simulation system indicated moderate levels of satisfaction, low to moderate feelings of presence, and acceptable system usability. In previous studies, such system-related factors have been shown to positively influence the overall VP interaction experience [40, 41, 46, 78]. However, their impact on the effectiveness of VP training has yet to be thoroughly examined. In our study, none of these variables significantly predicted improvements in the main outcome measures. One possible explanation might be that the moderate ratings of system-related factors may have diminished their observable impact on training gains. When system elements fulfill basic functional requirements but do not excel, their contribution to outcomes is likely to be limited, making pronounced effects less apparent. Participants’ open-ended system evaluation provided additional insights: Some participants criticized the restricted conversational depth, noting that the VP often responded with only single sentences. Others pointed to the rigid facial expressions, unnatural gestures, and monotonous voice, all of which potentially diminished the perceived realism of the interaction. Such limitations likely reduced the system’s ability to foster presence and authenticity, thereby constraining its potential training impact. Indeed, it has been shown that subtle design features, such as realistic head movements and facial expressions, play a key role in shaping the users’ sense of social presence [79]. More broadly, the evaluation indicates that the simulation content and design were not optimally aligned with participants’ individual needs, which were not systematically assessed prior to the intervention. This underscores the importance of participatory and user-centered design approaches in tailoring VP systems to support diverse learners [80, 81].

Our findings indicate that VP simulations hold considerable potential for psychotherapy training, yet significant opportunities for further optimization remain. First, assessing participants’ prior VP experience and training needs before the intervention allows designers to tailor scenarios to learners’ backgrounds, enhancing relevance and learning outcomes [81]. In our study, only two participants had prior VP experience, which may explain why some found the simulation demanding and confusing, as it did not align with their existing knowledge and skills.

Second, structured and tailored pre-briefings and debriefings are crucial for VP training, providing guidance, setting expectations, and supporting reflective learning [73]. In our study, the pre-briefing was limited to a brief written paragraph, and feedback was given automatically. As a result, participants likely did not benefit from the simulation as fully as possible, highlighting the need for more comprehensive, interactive preparatory and reflective elements to maximize learning outcomes.

Third, usability remains critical. Interfaces that are difficult to navigate increase cognitive load and distract learners from the training objectives. VP simulations for psychotherapy training should therefore be designed to support intuitive platform use and seamless workflows flow by providing step-by-step guidance for platform navigation and minimizing non-essential interface elements. Future research could measure cognitive load directly, for example, via the Multidimensional Cognitive Load Scale for Virtual Environments [82], and use eye-tracking [83] or think-aloud protocols [84] to capture learners’ real-time perceptions and uncover usability issues.

Lastly, given that AI-enabled VPs often provide unsolicited information, it is important to improve their response style and ensure that the VPs respond similarly to human patients to increase training fidelity and promote active, targeted engagement. Further enhancing the realism and immersion of VP environments, such as by using a videoconferencing interface instead of a generic simulated patient room, could more accurately reflect contemporary clinical practice, where psychotherapy is increasingly delivered online. Such adjustments may increase ecological validity and strengthen the perceived authenticity of the training experience.

Overall, by focusing on user-centered design, tailoring content to learners, and managing cognitive load, VP systems that are both effective and scalable for training complex clinical skills can be created [85].

Several limitations should be considered in the interpretation of the study results, offering directions for future research.

First, simulation-related variables for the control group were not assessed, which prevented direct comparisons between the two groups. Future studies should include equivalent measures across groups to allow more precise evaluations of VP-specific effects.

Second, participants were instructed to complete the intervention on four consecutive days, optimally not taking longer than a week. However, as the study was conducted online, adherence to this schedule could not be fully monitored. While most participants completed the intervention within seven days, the maximum duration observed was 24 days for one participant in the experimental group. Variations in completion timing, as well as other professional development opportunities, such as clinical supervision or other training, might have influenced the study outcomes. Future research could address this by more closely monitoring adherence or controlling for external influences between sessions.

Third, as feedback explained some of the variance in one subdimension of psychotherapeutic competence, it would be necessary to further study its effects in the future. Future research should therefore examine not only the presence of feedback but also its type and timing in order to clarify its specific role in VP training. Furthermore, personalized feedback, as a distinctive feature of AI-based VP simulation training, should be further refined and systematically studied.

Fourth, the questionnaires and videos were conducted in English. Only the VP simulation language was adjustable from English to German. English was chosen as the common language across conditions to ensure equivalence of the control material (video-based training) for both the German and UK sample. Thus, language-related anxiety [86] cannot be fully ruled out, but it likely did not affect the results beyond the general cognitive load of using a non-native language, as participants were explicitly informed beforehand which parts would be in English. Our sample consists of Master’s students and psychotherapists in training, who generally have high English proficiency, as psychology programmes in Germany set a minimum English language requirement for admission. From the beginning of their higher education, they are required to engage with English study materials, equipping them to understand and respond to the study content. In some cases, however, English proficiency could have affected comprehension and may also have contributed to the dropout rate of 33.8%, as some participants cited language difficulty as a reason for leaving.

Fifth, this study examined only a limited set of clinical conditions and a small number of relatively brief sessions. Future research should include more diverse patient cases and longer or additional VP sessions to provide a deeper understanding of VP interactions and training effectiveness. Given that participants reported the highest insecurity after interacting with the VP displaying suicidal tendencies, further exploration of such cases may be especially valuable [76]. Additionally, extending VP sessions to follow-up encounters beyond the diagnostic interview could also offer insights into longitudinal training effects.

Sixth, the current study may have been underpowered to detect interaction effects, indicating limited sensitivity to detect such effects. Accordingly, nonsignificant interactions should be interpreted with caution, and future studies with larger samples are needed to more conclusively examine potential moderators of VP training outcomes.

Finally, all competence-related outcomes relied on self-report measures. While self-reports are meaningful in capturing learners’ subjective experiences, they are limited in objectivity and may be affected by self-perception biases. Future research could complement self-report data with external evaluations, such as supervisor ratings, behavioral assessments by objective judges [87], or performance-based measures, to provide a more comprehensive picture of competence development.

In conclusion, AI-enabled VP training holds promise for enhancing psychotherapy training by providing interactive, hands-on learning experiences that can improve perceived clinical competence and reduce insecurities. To maximize this potential, VP systems need to adapt dynamically to learners’ prior knowledge, cognitive load, and individual training. Advancing AI-enabled VP simulations in this way represents a key opportunity for educational communities to develop scalable, effective tools that better prepare future psychotherapists and help meet the growing global demand for mental health services by smoothing the transition into clinical practice.

## Supplementary Information

Supplementary Material 1.

## Data Availability

Survey material, all data, and code can be found on OSF.

## References

[1] WHO. World Mental Health Report: Transforming mental health for all. 2022. https://www.who.int/publications/i/item/9789240049338

[2] Gilburt H, Mallorie S. Mental Health 360 | Workforce. https://www.kingsfund.org.uk/insight-and-analysis/long-reads/mental-health-360-workforce. Accessed 2025.

[3] Pakenham KI, Stafford-Brown J. Stress in clinical psychology trainees: current research status and future directions. Aust Psychol. 2012;47:147–55.

[4] Thériault A, Gazzola N. Feelings of inadequacy, insecurity, and incompetence among experienced therapists. Couns Psychother Res. 2005;5:11–8.

[5] Knox S, Callender KA, Mak TW, Skaistis S, Knowlton G. How graduate-student or recent graduate psychotherapists experience and manage errors in psychotherapy. Couns Psychol Q. 2022;35:397–420.

[6] Quinlan E, Schilder S, Deane FP. This wasn’t in the manual: a qualitative exploration of tolerance of uncertainty in the practicing psychology context. Aust. Psychol. 2021;56:154–167.

[7] Heinonen E et al. Psychotherapist trainees’ quality of life: patterns and correlates. Front Psychol. 2022;13:864691. 10.3389/fpsyg.2022.864691 PMC8988184 35401345

[8] Bell C, et al. The emotionally exhausted treating the mentally unwell? A systematic review of burnout and stress interventions for psychologists. Clin Psychol Psychother. 2024;31:e2909. 37691443 10.1002/cpp.2909

[9] Mcmahon A, Hevey D. It has taken me a long time to get to this point of quiet confidence: what contributes to therapeutic confidence for clinical psychologists? Clin. Psychol. 2017;21:195–205.

[10] Skovholt TM, Starkey MT. The three legs of the practitioner’s learning stool: practice, research/theory, and personal life. J Contemp Psychother. 2010;40:125–30.

[11] Stige SH, et al. Training students to become responsive therapists: implications from a sequential mixed-methods study on situations that therapists find challenging. BMC Med Educ. 2024;24:261. 38459480 10.1186/s12909-024-05236-1 PMC10924412

[12] Folkes-Skinner J, Elliott R, Wheeler S. A baptism of fire’: a qualitative investigation of a trainee counsellor’s experience at the start of training. Couns Psychother Res. 2010;10:83–92.

[13] Driscoll KA, Cukrowicz KC, Reitzel LR, Hernandez A, Petty SC, Joiner TEJ. The effect of trainee experience in psychotherapy on client treatment outcome. Behav Ther. 2003;341:165–77.

[14] Stein DM, Lambert MJ. Graduate training in psychotherapy: are therapy outcomes enhanced? J Consult Clin Psychol. 1995;63:182–96. 7751479 10.1037//0022-006x.63.2.182

[15] Swift JK, Greenberg RP. Premature discontinuation in adult psychotherapy: a meta-analysis. J Consult Clin Psychol. 2012;80:547–59. 22506792 10.1037/a0028226

[16] Frank HE, Becker-Haimes EM, Kendall PC. Therapist training in evidence-based interventions for mental health: a systematic review of training approaches and outcomes. Clin Psychol Publ Div Clin Psychol Am Psychol Assoc. 2020;27:e12330. 10.1111/cpsp.12330 PMC8174802 34092941

[17] Chernikova O, Heitzmann N, Stadler M, Holzberger D, Seidel T, Fischer F. Simulation-based learning in higher education: a meta-analysis. Rev Educ Res. 2020;90:499–541.

[18] Cook DA, Triola MM. Virtual patients: a critical literature review and proposed next steps. Med Educ. 2009;43:303–11. 19335571 10.1111/j.1365-2923.2008.03286.x

[19] Coyne E, Calleja P, Forster E, Lin F. A review of virtual-simulation for assessing healthcare students’ clinical competency. Nurse Educ Today. 2021;96:104623. 33125979 10.1016/j.nedt.2020.104623

[20] Shin S, Park JH, Kim JH. Effectiveness of patient simulation in nursing education: meta-analysis. Nurse Educ Today. 2015;35:176–82. 25459172 10.1016/j.nedt.2014.09.009

[21] Washburn M, Parrish DE, Bordnick PS. Virtual patient simulations for brief assessment of mental health disorders in integrated care settings. Soc Work Ment Health. 2020;18:121–48. 32952451 10.1080/15332985.2017.1336743 PMC7500537

[22] Plackett R, Kassianos AP, Mylan S, Kambouri M, Raine R, Sheringham J. The effectiveness of using virtual patient educational tools to improve medical students’ clinical reasoning skills: a systematic review. BMC Med Educ. 2022;22:365. 35550085 10.1186/s12909-022-03410-x PMC9098350

[23] Furuheim ACH, Lindenskov L. Virtual reality simulation as a learning activity for nursing students in mental health practice. Nurse Educ Pract. 2025;83:104272. 39892253 10.1016/j.nepr.2025.104272

[24] Jensen RAA, Musaeus P, Pedersen K. Virtual patients in undergraduate psychiatry education: a systematic review and synthesis. Adv Health Sci Educ. 2024;29:329–47. 10.1007/s10459-023-10247-6 37294380

[25] Foster A, Chaudhary N, Murphy J, Lok B, Waller J, Buckley PF. The use of simulation to teach suicide risk assessment to health profession trainees—rationale, methodology, and a proof of concept demonstration with a virtual patient. Acad Psychiatry. 2015;39:620–9. 25026950 10.1007/s40596-014-0185-9

[26] Nakash T, et al. Increasing resilience and preventing suicide: training and interventions with a distressed virtual human in virtual reality. Proc 22nd ACM Int Conf Intelligent Virtual Agents. ACM; 2022. p. 1–8. 10.1145/3514197.3549613.

[27] Pantziaras I, Fors U, Ekblad S. Training with virtual patients in transcultural psychiatry: do the learners actually learn? J Med Internet Res. 2015;17:e46. 25689716 10.2196/jmir.3497 PMC4376199

[28] Steenstra I, Nouraei F, Bickmore T. Scaffolding empathy: training counselors with simulated patients and utterance-level performance visualizations. Proc CHI Conf Hum Factors Comput Syst ACM. 2020;1–22. 10.1145/3706598.3714014.

[29] Yoo S, Kim S, Lee Y. Learning by doing: evaluation of an educational VR application for the care of schizophrenic patients. Ext Abstr CHI Conf Hum Factors Comput Syst. ACM; 2020. p. 1–6. 10.1145/3334480.3382851.

[30] Isaza-Restrepo A. The virtual patient as a learning tool: a mixed quantitative qualitative study. BMC Med Educ. 2018;18:297. 30522478 10.1186/s12909-018-1395-8 PMC6282259

[31] Kim GM, Lim JY, Kim EJ, Yeom M. Impact of virtual reality mental health nursing simulation on nursing students’ competence. J Multidiscip Healthc. 2024;17:191–202. 38226028 10.2147/JMDH.S435986 PMC10789577

[32] Sapkaroski D, Mundy M, Dimmock MR. Immersive virtual reality simulated learning environment versus role-play for empathic clinical communication training. J Med Radiat Sci. 2022;69:56–65. 34706398 10.1002/jmrs.555 PMC8892424

[33] Atuel HR, Kintzle S. Comparing the training effectiveness of virtual reality and role play among future mental health providers. Psychol Trauma Theory Res Pract Policy. 2021;13:657–64. 10.1037/tra0000997 33475410

[34] Novais F, Ganança L, Barbosa M, Telles-Correia D. Communication skills in psychiatry for undergraduate students: a scoping review. Front Psychiatry. 2022;13:972703. 36032255 10.3389/fpsyt.2022.972703 PMC9402997

[35] Stamer T, Steinhäuser J, Flägel K. Artificial intelligence supporting the training of communication skills in the education of health care professions. Scoping Rev J Med Internet Res. 2023;25:e43311. 10.2196/43311 PMC10337453 37335593

[36] Rudolph E, Engert N, Albrecht J. An AI-based virtual client for educational role-playing in the training of online counselors. Proc Int Conf Comp Supported Educ SCITEPRESS. 2024;108–17. 10.5220/0012690700003693.

[37] Shorey S, et al. Evaluation of a theory-based virtual counseling application in nursing education. CIN Comput Inf Nurs. 2023;41:385. 10.1097/CIN.0000000000000999 36728150

[38] Kakabayeva D, Abibulayeva A, Orazbayeva K, Naviy L. AI as a simulated client: impact on educational psychology students’ therapeutic skills. J Curric Stud Res. 2025;7:31–52.

[39] Aljohani AS, Karim Q, George P. Students’ satisfaction with simulation learning environment in relation to self-confidence and learning achievement. J Health Sci. 2016;4:228–35.

[40] Makransky G, Petersen GB. Investigating the process of learning with desktop virtual reality: a structural equation modeling approach. Comput Educ. 2019;134:15–30.

[41] Makransky G, Lilleholt L, Aaby A. Development and validation of the Multimodal Presence Scale for virtual reality environments: a confirmatory factor analysis and item response theory approach. Comput Hum Behav. 2017;72:276–85.

[42] Lv H, Ning Y, Li X, Meng L, Yang C, Jiang Y. Factors influencing medical students’ acceptance of clinical virtual simulation experiments: A combined TAM and TPB approach. IEEE Access. 2025;13:49801–16.

[43] Philip P, et al. Trust and acceptance of a virtual psychiatric interview between embodied conversational agents and outpatients. Npj Digit Med. 2020;3:2. 31934646 10.1038/s41746-019-0213-y PMC6946646

[44] Davids MR, Halperin ML, Chikte UME. Optimising cognitive load and usability to improve the impact of e-learning in medical education. Afr J Health Prof Educ. 2015;7:147.

[45] Lee KM, Presence. Explicated Commun Theory. 2024;14:27–50.

[46] Plotzky C, Lindwedel U, Bejan A, König P, Kunze C. Virtual reality in healthcare skills training: The effects of presence on acceptance and increase of knowledge. i-com. 2021;20:73–83.

[47] Lan YL, Chen WL, Wang YF, Chang Y. Development and preliminary testing of a virtual reality measurement for assessing intake assessment skills. Int J Psychol. 2023;58:237–46. 36720650 10.1002/ijop.12898

[48] Davis FD. Perceived usefulness, perceived ease of use, and user acceptance of information technology. MIS Q. 1989;13:319–40.

[49] Huang HM, Liaw SS. An analysis of learners’ intentions toward virtual reality learning based on constructivist and technology acceptance approaches. Int Rev Res Open Distrib Learn. 2018;19:91-115 .

[50] Green P, MacLeod CJ. SIMR: an R package for power analysis of generalized linear mixed models by simulation. Methods Ecol Evol. 2016;7:493–8.

[51] Kaplan AD, Cruit J, Endsley M, Beers SM, Sawyer BD, Hancock PA. The effects of virtual reality, augmented reality, and mixed reality as training enhancement methods: a meta-analysis. Hum Factors. 2021;63(4):706–26. 32091937 10.1177/0018720820904229

[52] Deutsche PsychotherpeutenVereinigung (DPtV). Report Psychotherapie 2021. 2021. https://www.dptv.de/fileadmin/Redaktion/Bilder_und_Dokumente/Wissensdatenbank_oeffentlich/Report_Psychotherapie/DPtV_Report_Psychotherapie_2021.pdf

[53] Jalali A, Harbi Houssein K, Fotsing S. Twelve practical tips for integrating AI into medical education: Tutorial to support educators across teaching, research, administration, and ethical domains. JMIR Med Educ. 2025;11:e81297. 41296915 10.2196/81297 PMC12743235

[54] Hettema J, Steele J, Miller WR. Motivational interviewing. Annu Rev Clin Psychol. 2005;1:91–111. 17716083 10.1146/annurev.clinpsy.1.102803.143833

[55] Imel Z et al. Design feasibility of an automated, machine-learning based feedback system for Motivational Interviewing. Psychotherapy 2019;56:318-328. 10.1037/pst0000221 PMC11270535 30958018

[56] Kang SHK. Spaced repetition promotes efficient and effective learning: policy implications for instruction. Policy Insights Behav Brain Sci. 2016;3:12–9.

[57] Melluish S, Crossley J, Tweed A. An evaluation of the use of simulated patient role-plays in the teaching and assessment of clinical consultation skills in clinical psychologists’ training. Psychol Learn Teach. 2007;6:104–13.

[58] Larson LM, Suzuki LA, Gillespie KN, Potenza MT, Bechtel MA, Toulouse AL. Development and validation of the Counseling Self-Estimate Inventory. J Couns Psychol. 1992;39:105.

[59] Tavakol M, Dennick R. Making sense of Cronbach’s alpha. Int J Med Educ. 2011;2:53–5. 28029643 10.5116/ijme.4dfb.8dfd PMC4205511

[60] Schmitt N. Uses and abuses of coefficient alpha. Psychol Assess. 1996;8:350-353 .

[61] Axboe MK, Christensen KS, Kofoed PE, Ammentorp J. Development and validation of a self-efficacy questionnaire (SE-12) measuring the clinical communication skills of health care professionals. BMC Med Educ. 2016;16:272. 27756291 10.1186/s12909-016-0798-7 PMC5069791

[62] ICD-10. https://icd.who.int/browse10/2019/en. Accessed 2026.

[63] Levett-Jones T, et al. The development and psychometric testing of the Satisfaction with Simulation Experience Scale. Nurse Educ Today. 2011;31:705–10. 21288606 10.1016/j.nedt.2011.01.004

[64] Brooke J. SUS - A quick and dirty usability scale. Usability Eval Ind. 1996;189:4–7.

[65] Bates D, Mächler M, Bolker B, Walker S. Fitting linear mixed-effects models using lme4. J Stat Softw 2015;67:1–48.

[66] Lenth RV. emmeans: Estimated Marginal Means. 2024. 10.32614/CRAN.package.emmeans.

[67] Lakens D. Sample Size Justification. Collabra Psychol. 2022;8:33267.

[68] Bedmutha MS, et al. ConverSense: An automated approach to assess patient-provider interactions using social signals. Proc CHI Conf Hum Factors Comput Syst ACM. 2024;1–22. 10.1145/3613904.3641998. 10.1145/3613904.3641998 PMC11175388 38872922

[69] Hersoug AG, Høglend P, Havik OE, Monsen JT. Development of working alliance over the course of psychotherapy. Psychol Psychother Theory Res Pract. 2010;83:145–59. 10.1348/147608309X471497 19796494

[70] Sommers-Flanagan J, Bequette T. The initial psychotherapy interview with adolescent clients. J Contemp Psychother. 2013;43:13–22.

[71] Kiesewetter J, et al. Learning clinical reasoning: how virtual patient case format and prior knowledge interact. BMC Med Educ. 2020;20:1–10. 10.1186/s12909-020-1987-y PMC7071577 32171297

[72] Liaw SY, Ooi SW, Rusli KDB, Lau TC, Tam WWS, Chua WL. Nurse-physician communication team training in virtual reality versus live simulations: randomized controlled trial on team communication and teamwork attitudes. J Med Internet Res. 2020;22:e17279. 32267235 10.2196/17279 PMC7177432

[73] Lee J, Kim H, Kim KH, Jung D, Jowsey T, Webster CS. Effective virtual patient simulators for medical communication training: a systematic review. Med Educ. 2020;54:786–95. 32162355 10.1111/medu.14152

[74] Sweller J. Cognitive Load Theory. Psychol Learn Motivation. 2011;55:37–76.

[75] Kalyuga S. Expertise reversal effect and its implications for learner-tailored instruction. Educ Psychol Rev. 2007;19:509–39.

[76] Bühlmann V, et al. Therapists’ emotional state after sessions in which suicidality is addressed: need for improved management of suicidal tendencies in patients with borderline personality pathology. BMC Psychiatry. 2021;21:590. 34814860 10.1186/s12888-021-03549-9 PMC8609732

[77] Kasapakis V, Dzardanova E, Agelada A. Virtual reality in education: the impact of high-fidelity nonverbal cues on the learning experience. Comput Educ X Real. 2023;2:100020.

[78] Olaussen C, Heggdal K, Tvedt CR. Elements in scenario-based simulation associated with nursing students’ self-confidence and satisfaction: a cross-sectional study. Nurs Open. 2020;7:170–9. 31871700 10.1002/nop2.375 PMC6917966

[79] Uhl JC, Schrom-Feiertag H, Regal G, Gallhuber K, Tscheligi M. Tangible immersive trauma simulation: Is mixed reality the next level of medical skills training? In Proc CHI Conf Hum Factors Comput Syst. ACM; 2023. p. 1–17. 10.1145/3544548.3581292.

[80] Yarmand M, Chen C, Cheng K, Murphy J, Weibel N. ‘I’d be watching him contour till 10 o’clock at night’: understanding tensions between teaching methods and learning needs in healthcare apprenticeship. In: Proc CHI Conf Hum Factors Comput Syst. ACM; 2025. p. 1–19. 10.1145/3613904.3642453.

[81] Zhu XT, Cheerman H, Cheng M, Kiami SR, Chukoskie L, McGivney E. Designing VR simulation system for clinical communication training with LLMs-based embodied conversational agents. Ext Abstr CHI Conf Hum Factors Comput Syst. ACM; 2025. p. 1–9. 10.1145/3706599.3719693.

[82] Andersen MS, Makransky G. The validation and further development of a multidimensional cognitive load scale for virtual environments. J Comput Assist Learn. 2021;37:183–96.

[83] Gao H, Gao Y, Kasneci E. An explainable machine learning approach for cognitive load detection in virtual reality using eye tracking data. Proc Int Conf Multimedia Retrieval. ACM; 2025. p. 340–8. 10.1145/3731715.3733275.

[84] Negrão MD, Ferreira W, Bohrer B, Freitas CMDS, Maciel A, Nedel L. Design and Think-aloud study of an immersive interface for training health professionals in clinical skills. symp virtual augmented reality. ACM; 2023. p. 157–65. 10.1145/3625008.3625037.

[85] Seiferth C, et al. How to e-mental health: a guideline for researchers and practitioners using digital technology in the context of mental health. Nat Ment Health. 2023;1:542–54.

[86] Matsuda S, Gobel P. Anxiety and predictors of performance in the foreign language classroom. System. 2004;32:21–36.

[87] Nouraei F et al. Virtual agent-based communication skills training to facilitate health persuasion among peers. In: Proc ACM Hum Comput Interact. ACM; 2025. p. 1–24. 10.1145/3711101.

