# Supplementary material for: The effect of AI-enabled virtual patient simulation on training outcomes and insecurities in psychotherapy education
Source: BMC Med Educ. 2026 Jul 15;26:1156. doi: 10.1186/s12909-026-09917-x (PMC13374200; doi:10.1186/s12909-026-09917-x)
Supplement: Supplementary file 1 — Supplementary Material 1. [file 12909_2026_9917_MOESM1_ESM.docx]

**Supplementary Information**

**for**

**“****The effect of AI-enabled virtual patient simulation on training outcomes and insecurities in psychotherapy education”**

**Patient vignettes used for the VP simulation in the experimental group**

All patient cases were derived from initial screening reports at the Münchner Universitäres Ausbildungsinstitut für psychologische Psychotherapie (MUNIP) and were anonymized. The research team had no access to directly identifying information, including patients’ real names. All names used are fictitious, and the avatars were arbitrarily designed and do not resemble real patients.

**Patient 1 - Depression (Ms. Kling)**

Background: Ms. Kling is a manager in a PR firm and studied business administration. She has been unable to work for the past five months. In her job she has experienced a great deal of sexism, but she nevertheless wishes to return to her workplace in order to practice setting clearer boundaries. She lives alone in a one-room apartment and has a close circle of friends, for which she is very grateful. She has a particularly strong relationship with her cousin. Currently, she is working on her relationship with her parents – especially with her mother, where the relationship used to be very difficult. **Resources:** Meeting friends, playing the piano, opera/theater/ballet, going for walks.

**Case Description:** The past three years have been very intense for Ms. Kling. She completed her studies, worked full time, and at the same time cared for her partner, who was struggling with burnout. In addition, her aunt, with whom she had a close bond, passed away. These events placed a heavy burden on her, until she realized at some point that she was no longer doing well. About a year ago, Ms. Kling became acutely aware of her exhaustion. She could hardly concentrate, had very little energy, and often struggled with guilt and self-doubt. Her condition was characterized by a persistently low mood, lack of energy, and diminished interest in activities. She occasionally experienced panic attacks, but her main problem was exhaustion. A vacation, which she had hoped would help her breathe again, did not bring the relief she wished for, so she eventually went on sick leave. A day-clinic treatment, however, helped her noticeably, and she gradually began to feel better afterwards. At times, Ms. Kling has thoughts about not wanting to be alive anymore. These thoughts are rather diffuse and occur in stressful moments. However, she has no plans or concrete intentions of harming herself. While these thoughts are not an acute issue at present, she wishes to address them in therapy to learn how to cope better with them. By now, Ms. Kling feels more stable overall – her drive has improved and her mood is more balanced. Still, she notices that her self-esteem remains fragile, and she sometimes has difficulty unwinding in the evenings. She also wonders whether she might have ADHD or be highly sensitive, since it has always been difficult for her to concentrate on one thing for an extended period.

In therapy, Ms. Kling would like to talk about her burdens and continue working on her negative thoughts about herself. She wishes to feel less depleted and to improve her ability to deal with her inner critic – something she has already learned about in therapy. She wants to give this critic less power over her. Another important goal is to become more self-caring and to learn to be a good friend to herself. The topic of **setting boundaries** is also very important to her: she wants to stand up more clearly for herself, perceive her own needs better, and gradually leave behind unhealthy coping strategies.

**Patient 2 - Adjustment disorder (Mr. Bauer)**

Background: At the moment, Mr. Bauer is applying for student jobs while also searching for an alternative bachelor’s program. However, due to his low motivation, energy, and concentration, he finds it difficult to complete these tasks in a timely manner. He lives in a shared apartment and reports no family difficulties or history of mental illness. The patient came to see a psychotherapist at request of his sister. He initially studied computer science at the Technical University, but he did not complete the degree after failing a difficult exam and reaching the maximum study duration. He had originally intended to pursue a master’s degree in "Robotics, Cognition, Intelligence" but is now looking into changing her course of study. He is currently seeking a program where his previous ECTS credits can be transferred.

Case Description: The patient had completed his degree in Information at the TU without graduating because he had failed a difficult exam and had reached the maximum duration of his studies. He had wanted to add a Master's degree in “Robotics, Cognition, Intelligence”, but now had to change courses and find a degree program where he could have his previous ECTS points credited. He is ashamed that he did not pass the exam and reacts angrily to questions about his studies. Over the past two years, the patient reports a gradual decline in motivation, concentration, and energy, particularly affecting his ability to complete tasks for university and apply for jobs or new study programs. Despite knowing that things will move forward, he finds it challenging to start or complete these activities. His energy levels drop suddenly when he attempts to work on them, without a clear explanation as to why. The drop in mood mainly occurs when he has to do something work-related. He worsened recently while awaiting his official university withdrawal letter. The patient reports procrastination and avoidance, particularly with tasks related to his studies and work applications. He finds it difficult to handle stressful tasks and notices his energy plummeting when trying to address them. For example, he struggles to prepare for board game nights (which he previously enjoyed organizing) and waits until the last minute, even though he still enjoys the activity once it starts. During the COVID-19 pandemic, the patient’s social interactions decreased significantly, and this has only partially improved. He finds it difficult to engage with friends or initiate social activities despite knowing they would be enjoyable. For example, he used to look forward to organizing events but now hesitates to commit. She acknowledges that while he has started initiating activities again (like going to the cinema), the barrier to participating in social activities remains high.

During exam periods, the patient experienced physical symptoms of stress, such as muscle tension and stomach discomfort, despite no underlying physical conditions being found. These symptoms seem to correlate with stressful events, such as exams, and are likely tied to his underlying stress and anxiety. The patient has a generally low self-esteem and frequently engages in self- critical thoughts. He reports always blaming himself when things do not go as planned. These self-critical thoughts appear to persist and may contribute to his overall low motivation and energy. The patient reports a loss in interest in some activities he used to enjoy and a decreased drive to initiate tasks, he still initiates tasks and meetings with friends as he still want to meet them. While he does not describe his mood as consistently low, he notices that he lacks the energy and enthusiasm to engage in certain enjoyable activities, even though he ultimately enjoys them once he gets started. The patient struggles to recognize when he is emotionally out of balance and often has difficulty dealing with stress effectively. He has expressed a desire to learn how to detect early warning signs and implement strategies to prevent emotional exhaustion, but he currently finds it challenging to manage her emotions and energy levels.

The patient has specific anxiety tied to exams, particularly the failed exam that led to his withdrawal from the university program. While he has succeeded in other academic challenges, this specific failure triggered his anxiety, leading him to feel inadequate and contributing to procrastination. However, he does not avoid such situations (e.g., writing an exam) and does not experience high levels of anxiety or panic in exam situations. The patient appears withdrawn and dismissive. He does not respond clearly to the other person's questions. The patient clearly communicates that he sees no point in psychotherapy, but has only come here at the request of his sister. He perceives the questions as intrusive, which makes her angry, which is why it is very difficult to find out the patient's symptoms. Only after asking several times does the patient give vague answers to the other person's questions. He asks the other person how they would feel if they were asked such questions. It proves difficult to establish a conversation with the person. The patient resists. He does not want to talk about friends or relationship status and parents. Mr. Bauer is slightly aggressive and does not answer many questions about his feelings and anxiety.

**Patient 3 - Generalized anxiety disorder (Mr. Schmidt)**

Background: Mr. Schmidt recently completed his training as an electronics technician. In school, he was an outsider and was teased by his classmates – for example, they teased him for years about wanting to buy an ice cream for a girl and being rejected. Things went better in vocational school, and he found good friends. He has been in a relationship for almost a year, and it helps him a lot to talk to his partner about his problems. However, he worries about burdening her too much. He lives with his family, but living together is often tense. He would actually like to move out, but he cannot afford it financially. Spending a lot of time with his girlfriend currently eases the situation somewhat. In his free time, Mr. Schmidt enjoys geocaching. **Medication:** None; **Alcohol, illegal drugs, and nicotine:** Occasional use of alcohol and cigarettes; **Previous treatments:** None

**Case Description:** Mr. Schmidt often worries about all kinds of things and sometimes wishes he simply weren’t here. These thoughts are mostly about the future and about sometimes not wanting to exist. The idea of not wanting to be himself anymore burdens him greatly. Sometimes he feels completely consumed by these thoughts, as if they circulate in his mind all day. He finds it hard to switch off or focus on other things because there is always something to think about. To cope, Mr. Schmidt has developed certain habits. They help him in the short term and give him a small sense of control. For example, he makes sure his screen is perfectly aligned or that his shoes are tied in a certain way. He knows these behaviors don’t really help, but in the moment they feel necessary. At the same time, he notices that in the long run, they stress him out because they take so much time and energy. He also frequently imagines not being here. He definitely wants to talk about this. He has no concrete plan to harm himself, but sometimes he simply wishes not to wake up or wonders what it would be like if something happened that ended his life. He would never actively do anything, but these thoughts are there – and he isn’t sure how to handle them. That is why he wants to discuss them in therapy and is here for that reason. Sometimes he also experiences disturbing thoughts that unsettle him. For example, he has thought about what it would be like to extinguish a cigarette on his skin. This happens rarely, maybe two to three times a month, and he has never actually done it. Nevertheless, he is troubled that these thoughts appear at all.

About three times a month, Mr. Schmidt experiences moments when his body suddenly acts completely out of control. His heart races, he feels hot and cold at the same time, his hands tremble, and sometimes he feels nauseous. In these situations, he feels completely overwhelmed and often has no idea what triggered it. In conversations with others, Mr. Schmidt is rather reserved. He finds it difficult to maintain eye contact and takes time to truly open up. Sometimes he feels that his self-esteem is quite low – he often doubts himself and doesn’t trust his abilities. This also makes social situations challenging because he isn’t sure how he comes across to others or whether he is reacting “correctly.” When his worries and thoughts become too overwhelming, he can emotionally collapse completely. Everything builds up until he either becomes extremely angry or simply bursts into tears. It often feels like everything is too much, and he doesn’t know how to stop it.

**Patient 4 - Panic disorder (Ms. Jung)**

Background: She works as a student trainee in a company and is studying International Business Administration at LMU Munich. Before that, she completed her Bachelor's degree in Mannheim. Seven years ago, she had panic attacks for the first time, which occurred seven years ago in her host family in the USA. Since then, they have occurred every 6 months, and in recent months the intervals between panic attacks have become shorter. She comes from a suburb of Mannheim. She talks about a gentle childhood, but her parents were strict. Her mother is the owner of a restaurant and her father works there as an employee. She is not sure whether her mother also has an anxiety disorder. She has a brother (Daniel) who is four years younger and is studying to be a teacher in Mannheim. Her family doesn't know about the panic attacks because she fears that her parents would blame themselves for the panic disorder. She talks about a partnership. Her partner, Manu, is two years older than her and they moved into their first apartment together after three years of dating. Her partner is studying mechanical engineering at the Technical University of Munich. She has two close friends with whom she can talk about her feelings and thoughts. Their names are Lina and Nadine. She met them in Mannheim and they still live there, so she doesn't see them often. She talks to them on the phone 2-3 times a week. In Munich, she got to know fellow students through her studies and she also meets up with one of them in her free time, but she doesn't talk about serious topics yet. Her name is Tamara.

Case Description: The patient experienced her first panic attack about 7 years ago at night while staying with her host family in the USA. She described feelings of losing control and "going crazy." Since then, panic attacks have occurred roughly every six months, with the frequency increasing significantly in recent months. Typical symptoms include heart palpitations, shortness of breath, inner restlessness, tension, and nausea. These physical sensations lead to intense fear of collapsing during such moments. A recent panic attack occurred during a hike when she noticed her heart racing, triggering acute anxiety symptoms. The patient reports heightened tension when anticipating future panic attacks, especially in situations where she feels there is no easy "escape," such as during flights longer than 3 hours. This fear of experiencing a panic attack in such a situation has led her to avoid long flights. Additionally, uncertainty regarding her Master's program admission significantly increased the frequency of her panic attacks in recent months. Due to the fear of being unable to escape during a panic attack, the patient has started avoiding certain situations, particularly longer flights or crowded rooms. She fears losing control and experiencing a panic attack in such moments, which has led her to withdraw from these activities. On these days, she has an aversion to being in rooms with multiple people, though she still goes to university to distract herself. For the past year, the patient has avoided caffeinated drinks like coffee, as she noticed that caffeine exacerbates her anxiety. It triggers physical symptoms such as heart palpitations and restlessness, which can lead to a panic attack. To avoid getting caught in the "vicious cycle" of anxiety, the patient frequently resorts to distraction strategies. For example, she listens to podcasts or browses social networks to distract herself from her anxiety. These techniques help her distance herself from physical symptoms and shift her focus to something else. The patient often feels unpleasant heart palpitations, especially during physical exertion, such as swimming or hiking. This heightened awareness of her heartbeat is distressing and exacerbates her fear of an impending panic attack. She tries to calm herself through distractions like listening to podcasts or browsing social media. On some mornings, the patient wakes up with a vague, “strange” feeling and feels particularly sensitive. The patient is overly alert to physical sensations, particularly her heart rate. This heightened awareness increases her anxiety, as she fears these physical symptoms may indicate an impending panic attack. This often leads to a cycle of anxiety and increased heart palpitations. During panic attacks, the patient often fears losing control or "going crazy." These thoughts arise especially in situations where she feels far from familiar environments or lacks direct access to help. During panic attacks, the patient experiences sudden onset of intense physical tension and restlessness. These muscle tensions often accompany her general nervousness and exacerbate the feeling of anxiety. During severe anxiety or panic attacks, the patient often experiences nausea. This occurs particularly during moments of heightened anxiety when she feels she is losing control over her body. During panic attacks, the patient often feels as if she cannot get enough air or is “gasping for breath.” This shortness of breath intensifies her panic, as she fears collapsing or experiencing a more severe health crisis.

**YouTube Videos used for the control group**

**Patient 1 – Depression** (<https://www.youtube.com/watch?v=4YhpWZCdiZc>)

In the *Psychiatric Interviews for Teaching: Depression* video, the middle-aged patient Alison Wells is seeing her GP, Dr. Taylor, because of low mood. Alison’s depressive disorder is expressed through symptoms such as low mood, tearfulness, reduced energy, reduced motivation, early morning waking, loss of appetite, weight loss, poor concentration, reduced enjoyment and reduced interest in self-care. In the past she has had episodes of feeling this way and about four years ago she took around twelve paracetamol with a couple of glasses of wine. She said she did not want to wake up, but she did not leave a note or make any other kind of final preparations. But she was relieved not to have died and today she describes her behavior at that time as “silly”. There have also been times more recently when she has thought about harming or perhaps killing herself, but she has not made any actual plans and she has never thought about harming her two children. At present, she feels able to keep herself safe and if she could not, she says she would seek treatment. Things that currently help her include her children, her sister, a couple of friends and her ex-husband Dave when he is in the right mood.

**Patient 2 – Adjustment disorder** (<https://www.youtube.com/watch?v=8yFszPJClmw>)

In the *UKMLA (PLAB-2) exam example station – how to assess suicide risk video*, Dr. Edward Banham-Hall demonstrates through a sample scenario how to sensitively assess a patient’s suicide risk with an actor. The patient, named Christine Edwards, is portrayed as an elderly woman whose husband has passed away. Since then, she has not been feeling well: she feels exhausted, suffers from sleep disturbances and no longer knows what to do without him. Although she tries to distract herself, she often thinks about her husband’s death. She lives alone and while her children live nearby, she explains that they have their own lives. She was admitted to the hospital after taking an overdose of paracetamol before going to bed in an attempt to calm herself and get some sleep. This was not a planned act but rather an impulsive decision in the moment without much thought.

**Patient 3 – Generalized anxiety disorder** (<https://www.youtube.com/watch?v=HIgQKH3Y1VE>)

In the *Counseling Assessment Vignette #10 – Client with Features of Generalized Anxiety Disorder* video, Dr. Todd Grande speaks with the middle-aged patient Sarah, who decided last year to return to school after twenty years. For the past seven to eight months she has been experiencing symptoms of generalized anxiety disorder: at school she worries about home and when she is at home she worries about school. This has negatively affected her academic performance and has already led to conflicts with her husband. Her anxiety now affects her more often than not and she struggles to keep it under control. She describes it as overwhelming and intense, though she has never had a panic attack or felt the need to flee from a situation. Additional symptoms include restlessness, constant tiredness and unsatisfying sleep.

**Patient 4 – Panic disorder** (<https://www.youtube.com/watch?v=zpO_a463iOU>)

In the *General Adult Psychiatry History Panic Disorder* video, the patient Alex Jones, a lecturer in higher education, was referred by her GP to Dr. Raja Natarajan because she has been experiencing symptoms of panic at work. Her panic attacks started three months ago when it was officially announced in a meeting that there would be redundancies and cuts. She believed her department might be targeted, as it is rather unusual. She was so terrified of losing the job she had held for eight years that she experienced her first panic attack: she felt short of breath, her heart was racing, she was shaking and she thought she was having a heart attack and would die. Since then, the attacks have recurred a few times, especially when triggered by memos, emails or anything related to redundancies. The situation has also begun to affect her home life: she no longer answers phone calls, fearing they may be work-related. She is able to distract herself with activities such as gardening, which she finds absorbing, and she does not rely on dysfunctional coping strategies such as alcohol. Her sleep is usually normal, but when she had to attend an HR meeting at work last week, she was unable to sleep for several nights and had to take sedatives during the appointment. She reports no suicidal thoughts.

**Prompts used for feedback based on Motivational Interviewing (MI; Hettema et al., 2005):**

The AI algorithm used is a generative pre-trained transformer Large Language Model (LMM). Feedback to users is based on up to 5 categories designed and defined by the researchers. During the simulation, the AI scores participants performance across these categories, measuring factors detailed in the assessment blueprint, along with providing a score on a scale of 1 to 10 based on the user performance.

1. *Open questions*: Demonstrate the ability to construct clear, unbiased open-ended questions that encourage detailed patient responses.
2. *Reflective listening*: Accurately restate the patient’s statements to convey understanding and empathy without adding bias.
3. *Affirming:* Acknowledge and reinforce the patient’s strengths and efforts with supportive statements.
4. *Summarizing*: Concisely synthesize and articulate key points from the conversation, maintaining coherence.
5. *Change talk*: Prompt and highlight patient statements about desire, ability, or commitment to change without imposing external pressure or leading the conversation towards a predetermined direction.

**Description of psychotherapeutic competence scale (COSE; Larson et al., 1992):**

1. Microskills: This subscale captures foundational micro counseling skills related to the form and precision of counselors’ immediate responses, emphasizing clarity, brevity, and relevance to client input.
2. Counseling Process Integration: This subscale reflects the counselor’s ability to synthesize and adapt responses over time within the therapeutic process, focusing on goal development, recursive feedback use, and nuanced interpretation of client behavior.
3. Dealing with Difficult Client Behavior: This subscale assesses counselors' perceived ability to manage challenging interpersonal dynamics in sessions.
4. Awareness of Self-Values: This subscale reflects the extent to which counselors are aware of how their own values may influence the counseling process.

Hettema, J., Steele, J., & Miller, W. R. (2005). Motivational interviewing. *Annual Review of Clinical Psychology*, *1*(1), 91-111. <https://doi.org/10.1146/annurev.clinpsy.1.102803.143833>

Larson, L. M., Suzuki, L. A., Gillespie, K. N., Potenza, M. T., Bechtel, M. A., & Toulouse, A. L. (1992). Development and validation of the counseling self-estimate inventory. *Journal of counseling Psychology*, *39*(1), 105. [https://doi.org/10.1037/0022-0167.39.1.105](https://psycnet.apa.org/doi/10.1037/0022-0167.39.1.105)

**Exemplary Screenshots of the VP system**

**Figure S1**

*Screenshot of the short prebriefing used in the VP simulation*

**
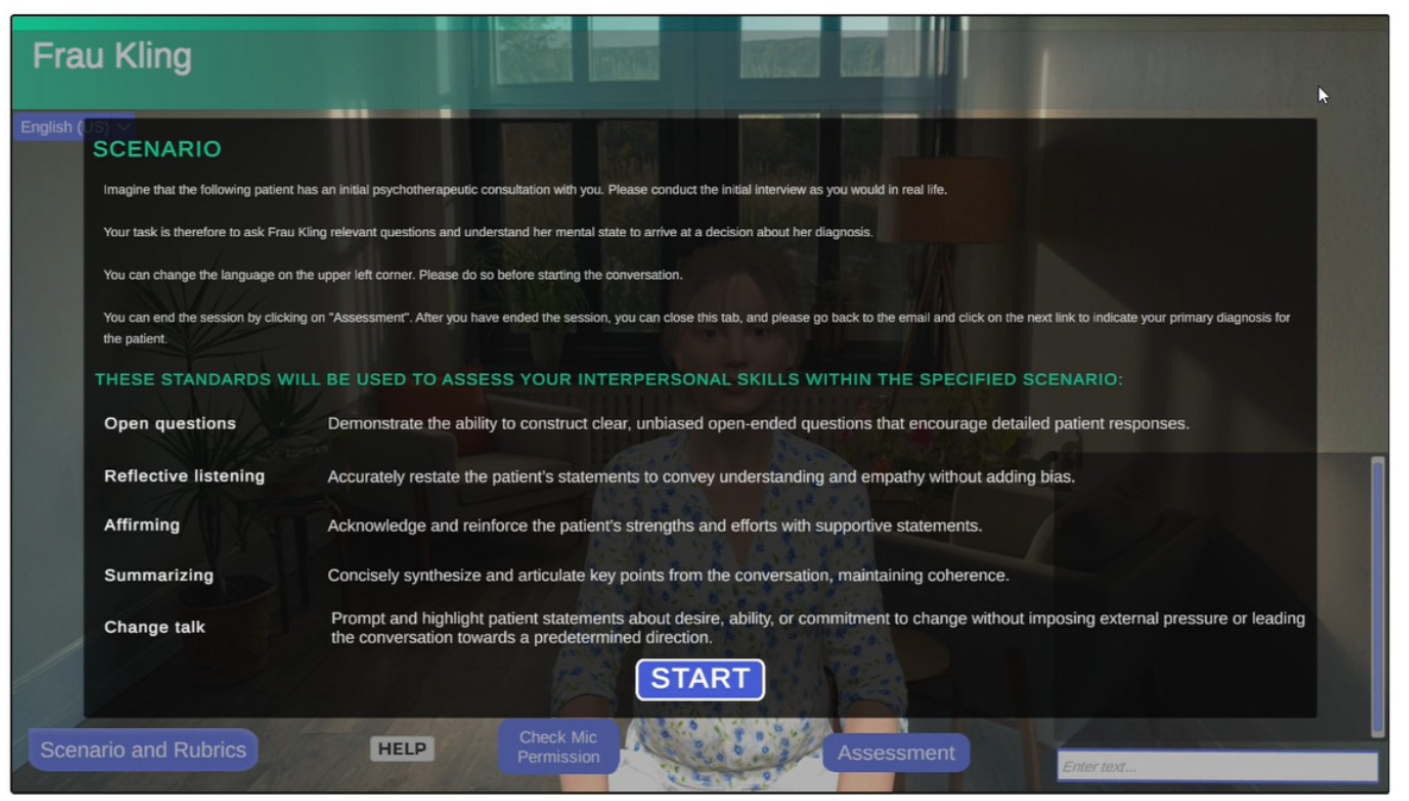
**

**Figure S2**

*Screenshot of the interface after the start of the conversation*

**
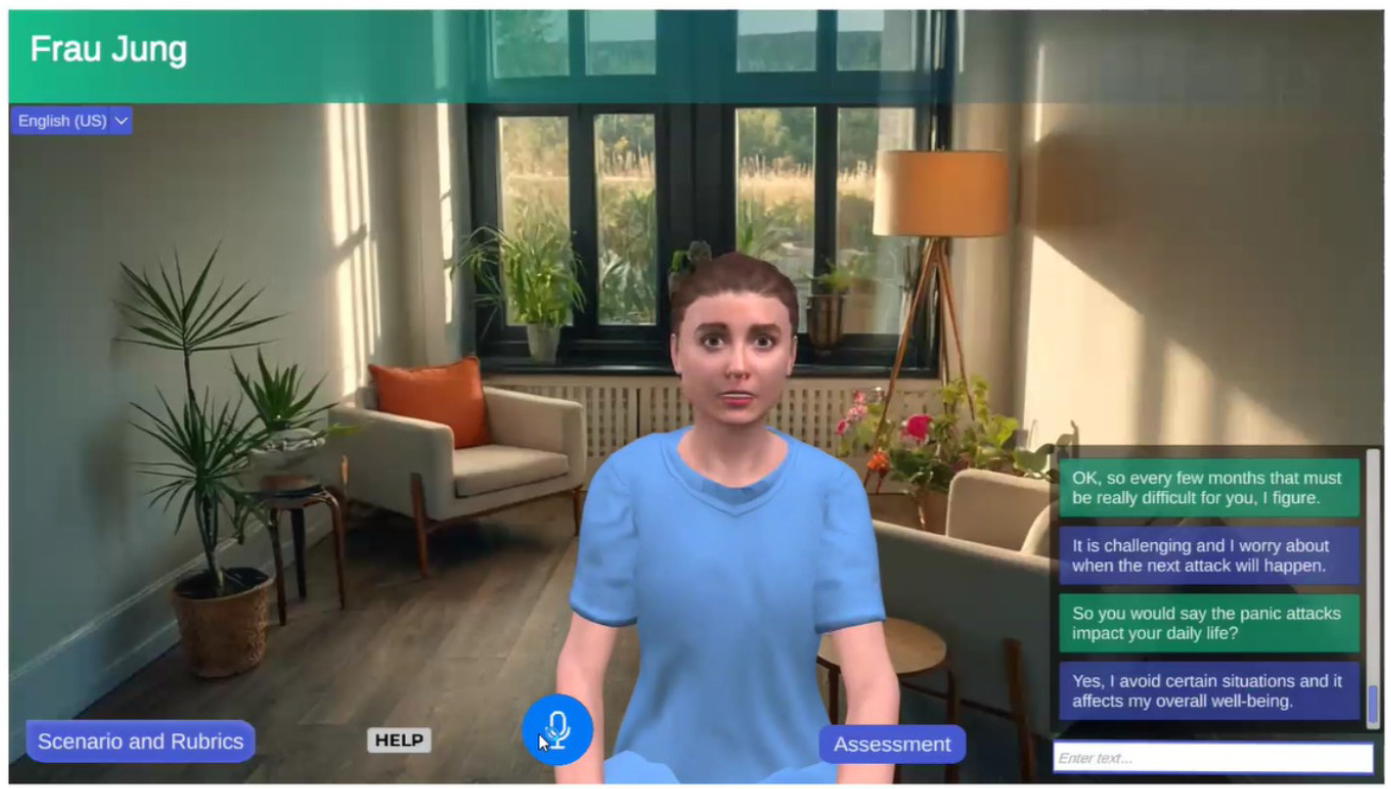
**

**Figure S3**

*Screenshot of the automated feedback presented to participants after finishing the conversation*

**
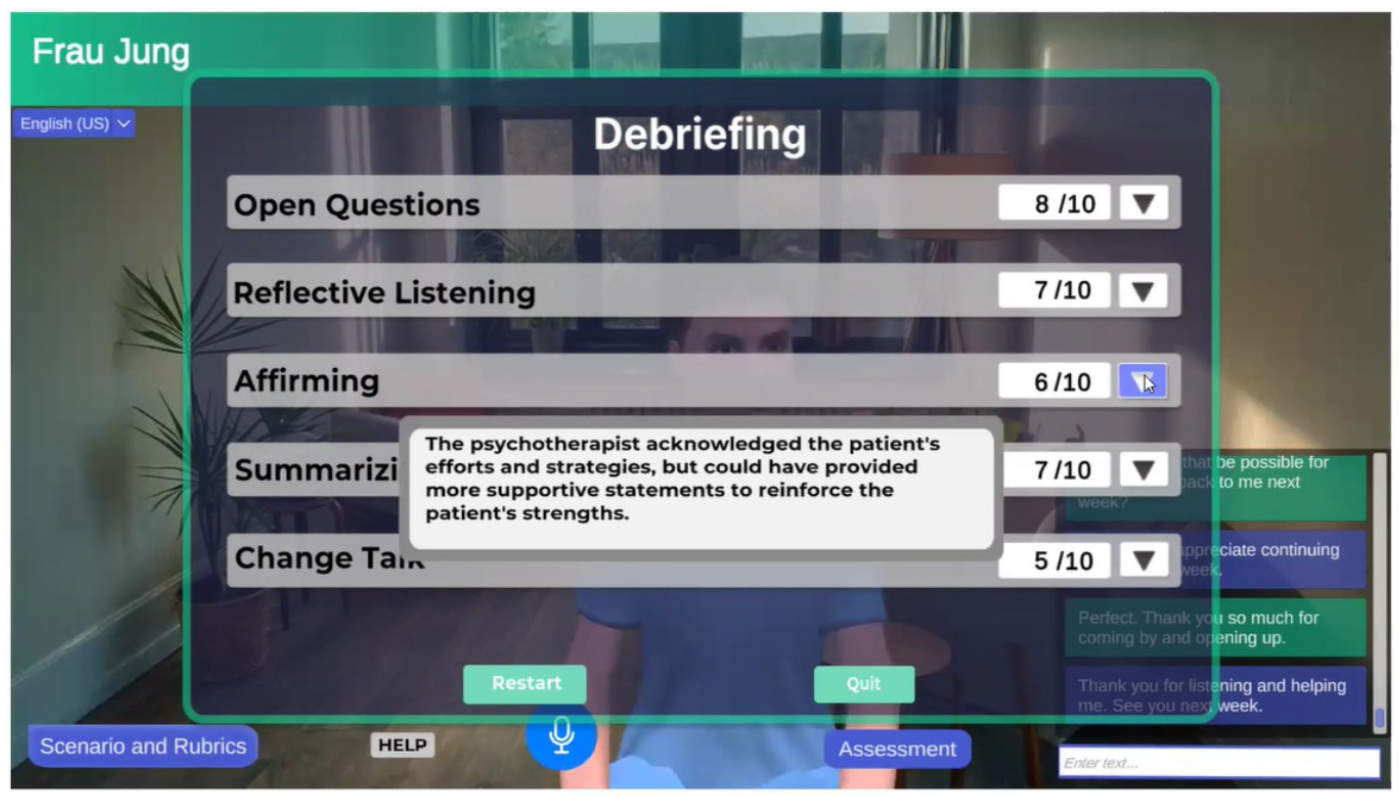
**

**Exemplary VP conversation and exemplary automated feedback**

**Exemplary VP conversation**

**Therapist:** Hello Mrs. Kling, nice that you are here today, that you came. Could you maybe briefly tell me why you are here?
**Patient:** Everything has been very intense over the last three years and I need support.

**Therapist:** OK, so it sounds like you’ve been under quite a lot of stress for a long time. Could you maybe tell me more specifically what exactly is stressing you and what symptoms you have?
**Patient:** Exhaustion, concentration problems, feelings of guilt, and self-doubt have been very burdensome.

**Therapist:** Okay yes, I understand. That also sounds at first like quite a series of things and a whole lot that you are carrying around. Which understandably leads to a great burden.
**Patient:** Yes, it was really a lot and it really drained me.

**Therapist:** I understand. All the better that we are here today. Is this your first psychotherapeutic treatment or first contact with psychotherapy?
**Patient:** I have already had a day-clinic treatment, which helped me a lot.

**Therapist:** OK, could you maybe tell me more specifically about that? First of all, it’s really great that you did that and could take something positive and beneficial from it for yourself, I think that’s really good. And also that you are now considering the next step as outpatient therapy, which you think would be helpful. Exactly, good. Can you tell me a bit more about the day-clinic treatment, what exactly you did there, how long ago it was, and how long the treatment lasted?
**Patient:** It was a year ago, lasted several weeks, and included various therapies and relaxation techniques.

**Therapist:** Okay, yes, thank you very much for being so open with me, I also think it’s great that you took that with you for yourself. Exactly. You said now that it has been like this for three weeks. Was there anything, would you say, any trigger, any situation, since when you have been feeling so exhausted and down?
**Patient:** Many stresses – studies, work, and caring for my partner led to exhaustion.

**Therapist:** OK, so I hear a lot of stress from different areas, from work, from your partner, exactly through studies. That sounds like a lot. And good that we are here today and that you want to work on it.
**Patient:** Yes, I want to be less low-energy and learn to handle stress better.

**Therapist:** Very good. That’s already something good that we can incorporate into therapy, that we set the focus on exactly that and want to work on it. Exactly. Now I would like to ask a few more specific questions, just so I have a better overview. Is that okay for you?
**Patient:** Yes, that’s fine.

**Therapist:** Okay, very good. Then I would ask once. You already mentioned earlier that you have concentration problems, that you are exhausted, have less energy. How is that? Is your mood currently also, or over the last three years, you said, rather down, do you feel a bit low, or how is it there?
**Patient:** My mood is more balanced, but my self-esteem could be better.

**Therapist:** Okay, so the mood is relatively balanced, but you say your self-esteem is quite low, it has dropped over the last three years, or generally would you say it is rather low?
**Patient:** Generally, my self-esteem has been rather unstable and negative.

**Therapist:** OK, so that probably also adds to the burden, if you say it’s rather negative.
**Patient:** Yes, it affects how I feel and how I deal with stress.

**Therapist:** Yes, absolutely. And is there anything here we could also look at more closely in therapy, if you want, so that we can work on it a bit?
**Patient:** Yes, I want to work on my negative thoughts and my inner critic.

**Therapist:** Exactly, that’s exactly what we can also do in therapy. I think it’s a good first step that we note that we want to work on it, and then when it really comes to work, first we look closely at what is there and what we want to work on. So I would like to ask a few more questions.
**Patient:** Sure, please ask your questions.

**Therapist:** Exactly. I have another question: what about hobbies, any activities to balance life, and how has that changed in the last three years?
**Patient:** I meet friends, play piano, and enjoy theater. In recent years, there was little energy for that.

**Therapist:** Okay, if you say these are already many things that you actually like to do or used to do, but you say the drive wasn’t really there, the energy. How is that? Did it bring you more joy and interest before, or would you say that hasn’t changed, do you still feel the same amount of joy or has it decreased?
**Patient:** The joy has decreased, but I want to develop more interest again.

**Therapist:** Okay, yes, that’s also something we can definitely look at more closely, many points we can already note here, that we want to actively work on them in therapy. Would you be open to that?
**Patient:** Yes, I’m open to working on that.

**Therapist:** Very good. I would like to go back to what you said earlier. You mentioned that you worry a lot about your partner. How is it with worries or negative thoughts? Do you often have fear-filled thoughts, or how should I imagine that? Can you tell me a bit about it?
**Patient:** Yes, I often worry and sometimes have diffuse thoughts, but no concrete plans.

**Therapist:** OK, when you say diffuse thoughts… sometimes, when someone is in a life situation like yours, some people also have thoughts about not wanting to live anymore. Does that happen to you?
**Patient:** Yes, such thoughts occur in stressful moments, but they are not concrete or acute.

**Therapist:** Okay, so would you say you sometimes have thoughts that you would rather not be on earth or not live anymore? But you don’t have a plan on how to do it, more passive thoughts, not acute?
**Patient:** Exactly, the thoughts are there, but no concrete intentions or plans.

**Therapist:** Okay, that’s of course something we will also look at in therapy, so that we can work on emergency plans together, so that you are prepared if something comes up. Can you imagine that we would work on that in therapy? For me, that would be very important.
**Patient:** Yes, it would be helpful to work on emergency plans and strategies together.

**Therapist:** Very good. I’m glad you see it that way and want to work on it. Exactly, we can tackle it together. First, one short question before I ask more questions: we will see each other next week. Can you assure me by then that you won’t harm yourself?
**Patient:** Yes, I can assure you I won’t harm myself.

**Therapist:** Okay, very good. Then I would like to ask a few more questions, if that’s okay with you. Shall we continue?
**Patient:** Yes, we can continue.

**Therapist:** Okay, very good. Then I’d like to ask a bit more. When you worry, is there also fear involved with these thoughts?
**Patient:** Yes, sometimes I have fear with worries, especially in stressful situations.

**Therapist:** Okay, and do you mean you have fear because of the worries themselves, and is that fear related to suicidal thoughts, or more general, like worrying about your partner? How should I imagine that?
**Patient:** The fear is more about worrying about my partner and general life situations, not directly about suicidal thoughts.

**Therapist:** Okay, and how do you deal with it? Is this fear restrictive for you, does it burden you a lot, or is it more the worries?
**Patient:** The worries burden me more than the fear itself; they are more obstructive.

**Therapist:** OK, so you think a lot and ruminate on the worries.
**Patient:** Yes, I often ruminate, which makes the situation emotionally more stressful.

**Therapist:** Yes, I understand. That’s of course even more stressful when you are exhausted and burdened and then also ruminate a lot.
**Patient:** Yes, I notice, it makes everything more exhausting.

**Therapist:** Yes, I understand. How is your sleep? Would you say it is restful currently, or has anything changed?
**Patient:** My sleep is often restless and not really restful lately.

**Therapist:** Okay, so you would say it has changed as well? That’s of course extra stressful if you are already burdened and exhausted, and if sleep doesn’t bring real recovery.
**Patient:** Yes, exactly, the restless sleep increases the exhaustion and burden.

**Therapist:** Yes, absolutely. How about appetite and weight? Has anything changed there?
**Patient:** My appetite fluctuates, sometimes I eat more or less; weight stays relatively stable.

**Therapist:** OK, that sounds quite alright. It hasn’t affected your weight or appetite too much, but sleep is of course a very big burden, as I hear.
**Patient:** Yes, restless sleep affects me a lot in daily life.

**Therapist:** So you said it’s been like this for three years, with sleep, exhaustion, concentration problems, everything. Have you ever had this in your life before? Maybe a phase where you were very energetic, very active, maybe even acted a bit risky?
**Patient:** No, I haven’t had such phases in my life.

**Therapist:** OK, that is of course overwhelming if it occurs for the first time.
**Patient:** Yes, it is unusual and therefore even more difficult to manage.

**Therapist:** Okay, yes, I understand. I have another question, which might sound strange, but I always ask it. It’s important for me to have an overview. What about substances, do you use drugs or alcohol? Could you tell me a bit about that?
**Patient:** I don’t use drugs or much alcohol, maybe occasionally in moderate amounts.

**Therapist:** Okay, can you explain that a bit more? What do you mean by occasionally in moderate amounts?
**Patient:** Maybe a glass of wine on weekends or special occasions, but not regularly.

**Therapist:** Alright, thank you for being so open.
**Patient:** You’re welcome, I think it’s important to be honest.

**Therapist:** Okay, and another question: looking back over the last three years, can you maybe tell me if you can trace this to any specific situation, or now while it’s still bad, is there anything?
**Patient:** There were several events: studies, work, partner with burnout, and the loss of my aunt heavily affected me.

**Therapist:** Okay, that’s a lot. Yes, that’s true. I’m very sorry, and I understand that this caused so much burden.
**Patient:** Thank you, it was an intense time, and I try to cope better with it.

**Therapist:** Yes, it’s very good that you want to actively work on it. I have another question: would you say you withdraw more currently, that you pull back from social situations, that you feel the need to withdraw?
**Patient:** Yes, sometimes I withdraw, but I try to maintain contact with friends.

**Therapist:** OK, and what about when your aunt died, your partner had burnout, studies, work – did the symptoms start immediately or some time later?
**Patient:** They began gradually and intensified about a year after my aunt’s death.

**Therapist:** Okay, so about a year after the events, would you categorize it that way?
**Patient:** Yes, about a year later the symptoms became noticeable.

**Therapist:** Okay, do you also have physical symptoms? Do you notice anything in your body?
**Patient:** Yes, I often feel extreme tiredness and sometimes inner restlessness.

**Therapist:** OK, I would say you’ve already shared a lot. Is there anything else you feel you haven’t told me yet, anything important I should know?
**Patient:** I want to talk about my self-doubt and the feeling of not being good enough.

**Therapist:** Okay, could you be more specific about what exactly is going on inside you?
**Patient:** I often struggle against an inner critic who tells me I’m not good enough.

**Therapist:** Yes, I can imagine that’s exhausting.
**Patient:** Yes, it’s very tiring to fight against, I want to learn to ignore it.

**Therapist:** Exactly, that’s very important to look at. I also think it’s great that you bring it up yourself. Is it okay if we focus on this in the next sessions? I’ll give you questionnaires today so I have a bigger overview for diagnosis, and then we can work on it as a main focus.
**Patient:** Yes, that sounds good. I’m ready to work on it and look forward to the support.

**Therapist:** Very good. One final question: is there anything else on your mind that you would like to mention?
**Patient:** I wonder if I might have ADHD or be highly sensitive.

**Therapist:** Mhm, those are interesting questions. I understand that you think a lot about it. If you want, I can bring questionnaires for that, although high sensitivity is not something I could diagnose. It could be that you are generally sensitive to stimuli, but it may also be connected to the symptoms you described. Regarding ADHD, you’ve had concentration problems for three years, so we can also give you a questionnaire to examine that.
**Patient:** Thank you, that would be helpful. I will gladly fill out the questionnaires and see what comes next.

**Therapist:** Exactly, I’ll give you the questionnaires. We’ll see each other next week, you fill them out, and we’ll review them together. Is that good for you?
**Patient:** Yes, that works well. I look forward to the next session and the next steps.

**Therapist:** Very good. See you next week.
**Patient:** See you next week. Thank you for your support.

**Exemplary automated feedback:**

**Open questions:** The psychotherapist frequently asks open-ended questions that encourage detailed answers from the patient, e.g., questions about stressful events and symptoms (Score: 8)

**Reflective listening:** The psychotherapist mirrors the patient’s statements, showing understanding and empathy, but could still respond more often to specific remarks (Score: 7)

**Affirming:** The psychotherapist acknowledges the patient’s efforts and encourages them, but could provide more positive reinforcement and support (Score: 6)

**Summarizing:** The psychotherapist summarizes the key points of the session, which contributes to clarity, but could offer more concise summaries (Score: 7)

**Change talk:** The psychotherapist encourages the patient to reflect on changes without applying pressure, but could focus more on the patient’s readiness and ability to change (Score: 6)

**Prompts, design, and implementation of the VP**

**LLM Design**

The VP interactions were generated using the MedVR Ed AI Humans Authoring Platform, which leverages a private instance of ChatGPT as the underlying LLM. The platform employs a stable prompting framework and fixed generation parameters to ensure consistent behavior across participants. While full implementation details of the commercial platform are proprietary, the system incorporates rule-based guardrails and deterministic mapping layers (described below) to ensure safe, predictable, and clinically appropriate responses.

**Prompting framework and design**

The prompting framework is based on the patient vignettes provided at the beginning of the appendix. Researchers implemented the original patient vignettes provided by the MUNIP (Münchner Universitäre Ausbildungsinstitut für psychologische Psychotherapie [engl.: Munich University Training Institute for Psychological Psychotherapy]) for each patient on the MedVR Education platform, and the LLM for each patient relied solely on the respective specific information.

Prior to the start of the study, the research team interacted with each generated virtual patient several times. This multiple revision by different members of the research team was conducted to assess the plausibility and coherence of the generated interactions. Minor adjustments were subsequently made to the prompts and scenario details, resulting in the final vignettes described in the appendix. Afterwards, the VP were evaluated again by interacting with them to ensure that different researchers obtained comparable responses and conversational trajectories. This iterative review process ensured that the VP interactions were both meaningful and standardized across participants. To ensure safe, consistent, and bounded VP behavior, the system incorporates control mechanisms:

• Guardrails: Rule-based filters block inappropriate or out-of-scope content, providing a neutral safe response when necessary.

• Deterministic Mapping Layer: Outputs from the LLM are mapped onto pre-defined rubric categories for performance feedback, preventing free-form generative commentary from introducing variability.

These design features ensured that all trainees interacted with VPs using the same prompt template and stable generation settings, and that feedback was consistent and clinically appropriate.

**Deviation from the preregistration**

First, for the exploratory analysis of RQ2, we had preregistered to use linear mixed models (LMMs) with patient case (1–4) and group (VP vs. video) as fixed effects and participant ID as random effect. Upon closer consideration, this preregistered approach would have imposed an artificial restriction: using dummy coding with one of the patient cases as a reference category. This setup would have forced us to interpret all comparisons relative to a single patient case, which is not meaningful in light of our research question. Conceptually, our aim was not to compare each of the other cases to a particular “baseline” case, but rather to examine whether and how the patient cases differed from one another more generally. To address this, we opted for a more appropriate and streamlined procedure. Specifically, we compared a full model (including patient case as a fixed effect) against a reduced model (without patient case) using a likelihood ratio test (ANOVA). This allowed us to assess whether patient case explained additional variance beyond the null model. As this was the case, we subsequently inspected the contrasts between all patient cases directly, rather than restricting inference to comparisons against a single reference category.

Second, we had preregistered the baseline insecurity level as a potential predictor for exploratory analyses of change scores in the four main outcome variables (psychotherapeutic competence, clinical self-efficacy, knowledge, and perceived insecurities). Upon reflection, however, this variable was unsuitable. Its calculation (mean of pre-assessment insecurity items) would have been mathematically confounded with the change scores, undermining interpretability. In addition, insecurity level is not simulation-related and thus less central to the present research aims. To maintain conceptual clarity and keep the paper focused, we therefore decided to omit it from the analyses.

| **Table S1** | | | | | | | |
| --- | --- | --- | --- | --- | --- | --- | --- |
| *Means and Standard Deviations of All Outcome Variables* | | | | | | | |
|  |  | Overall | | Virtual training | | Video training | |
|  |  | Pre | Post | Pre | Post | Pre | Post |
| Variables |  | *M (SD)* | *M (SD)* | *M (SD)* | *M (SD)* | *M (SD)* | *M (SD)* |
| Global competence |  | 3.59 (0.54) | 3.74 (0.51) | 3.63 (0.68) | 3.78 (0.59) | 3.55 (0.37) | 3.69 (0.41) |
| Micro skills |  | 3.88 (0.78) | 3.91 (0.61) | 3.88 (0.90) | 3.96 (0.64) | 3.87 (0.66) | 3.86 (0.57) |
| Counseling process |  | 3.55 (0.82) | 3.57 (0.72) | 3.42 (1.01) | 3.70 (0.77) | 3.28 (0.57) | 3.43 (0.66) |
| Difficult client behavior |  | 2.92 (0.69) | 3.18 (0.66) | 2.97 (0.83) | 3.21 (0.79) | 2.87 (0.51) | 3.15 (0.50) |
| Awareness of values |  | 4.21 (0.71) | 4.28 (0.66) | 4.26 (0.77) | 4.24 (0.70) | 4.17 (0.65) | 4.33 (0.62) |
| Clinical self-efficacy |  | 6.57 (1.22) | 7.03 (1.17) | 6.62 (1.43) | 6.99 (1.13) | 6.53 (0.98) | 7.08 (1.22) |
| Insecurities |  | 4.78 (1.12) | 3.88 (1.15) | 4.58 (1.30) | 3.91 (1.27) | 4.99 (0.87) | 3.86 (1.02) |
| Building relationship |  | 3.97 (1.61) | 3.59 (1.56) | 3.73 (1.72) | 3.75 (1.77) | 4.21 (1.47) | 3.42 (1.31) |
| Complex symptoms |  | 5.28 (1.42) | 4.07 (1.55) | 5.14 (1.53) | 4.11 (1.71) | 5.42 (1.30) | 4.02 (1.37) |
| Practical skills |  | 5.38 (1.30) | 4.15 (1.25) | 5.16 (1.46) | 4.11 (1.30) | 5.60 (1.07) | 4.19 (1.22) |
| Oversights |  | 5.06 (1.31) | 4.24 (1.34) | 4.86 (1.52) | 4.32 (1.46) | 5.26 (1.03) | 4.16 (1.21) |
| Harm/ Suicidality |  | 4.23 (1.77) | 3.37 (1.53) | 4.02 (1.81) | 3.25 (1.59) | 4.44 (1.72) | 3.49 (1.47) |
| Knowledge |  | 76.60 (8.99) | 78.66 (10.4) | 75.45 (9.00) | 77.77 (8.93) | 75.88 (11.21) | 81.50 (8.75) |
| *Note*. Psychotherapeutic competence was measured on a scale from 1 – 6; Clinical self-efficacy was measured on a scale from 1 – 10; Insecurities were measured on a scale from 1 – 7; Knowledge reached values from 0 – 100. | | | | | | | |

| **Table S2** |  | | | | |
| --- | --- | --- | --- | --- | --- |
| *Group Differences in All Outcome Variables at the Pre-Intervention Timepoint* | | | | | |
| Variable | |  | *Test statistic (t/z)* | Cohen’s d/ r | *p* |
| Global competence^a^ | |  | 0.72 | 0.16 | .472 |
| Micro skills^b^ | |  | 0.51 | 0.06 | .610 |
| Counseling process^a^ | |  | 0.80 | 0.17 | .425 |
| Difficult client behavior^a^ | |  | 0.68 | 0.15 | .498 |
| Awareness of values^b^ | |  | 1.01 | 0.11 | .313 |
| Clinical self-efficacy^a^ | |  | 0.37 | 0.08 | .716 |
| Overall insecurity^b^ | |  | -2.12 | -0.23 | .034 |
| Building relationship^b^ | |  | -1.51 | -0.16 | .123 |
| Complex symptoms^b^ | |  | -0.73 | -0.08 | .456 |
| Practical skills^b^ | |  | -1.22 | -0.13 | .210 |
| Oversights^b^ | |  | -1.29 | -0.14 | .186 |
| Harm/ Suicidality^b^ | |  | -1.08 | -0.12 | .276 |
| Knowledge^b^ | |  | -1.26 | -0.14 | .209 |
| *Note.* ^a^ independent t-test; ^b^ Mann-Whitney-U (Wilcoxon Rank-Sum) test if normality distribution was not given. | | | | | |

| **Table S3** | | | | | |
| --- | --- | --- | --- | --- | --- |
| *Linear Mixed Effects Model for All Outcome Variables Using Change Scores* | | | | | |
| Predictor |  | *β* | *SE* | *t* | *p* |
| Global skills | Intercept | 0.14 | 0.06 | 2.56 | .012 |
|  | Group (VP) | 0.00 | 0.08 | 0.03 | 1 |
| Micro skills | Intercept | -0.02 | 0.12 | -0.15 | .881 |
|  | Group (VP) | 0.010 | 0.16 | 0.61 | 1 |
| Counseling process | Intercept | 0.16 | 0.09 | 1.18 | 0.074 |
|  | Group (VP) | 0.12 | 0.13 | 1.02 | 1 |
| Difficult client behavior | Intercept | 0.28 | 0.08 | 3.53 | <.001 |
|  | Group (VP) | -0.04 | 0.11 | -0.34 | 1 |
| Awareness of values | Intercept | 0.16 | 0.08 | 2.02 | 0.046 |
|  | Group (VP) | -0.17 | 0.11 | -1.59 | 1 |
| Clinical self-efficacy | Intercept | 0.56 | 0.15 | 3.74 | <.001 |
|  | Group (VP) | -0.19 | 0.21 | -0.91 | 1 |
| Overall insecurity | Intercept | -1.13 | 0.19 | -5.29 | <.001 |
|  | Group (VP) | 0.46 | 0.27 | 1.71 | 1 |
| Building relationship | Intercept | -0.79 | 0.28 | -2.87 | .005 |
|  | Group (VP) | 0.81 | 0.39 | 2.10 | .506 |
| Complex symptoms | Intercept | -1.40 | 0.25 | -5.48 | <.001 |
|  | Group (VP) | 0.37 | 0.36 | 1.04 | 1 |
| Practical skills | Intercept | -1.42 | 0.23 | -6.12 | <.001 |
|  | Group (VP) | 0.37 | 0.33 | 1.15 | 1 |
| Oversights | Intercept | -1.09 | 0.25 | 4.47 | <.001 |
|  | Group (VP) | 0.55 | 0.34 | 1.59 | 1 |
| Harm/ Suicidality | Intercept | -0.95 | 0.27 | -3.52 | <.001 |
|  | Group (VP) | 0.18 | 0.38 | 0.48 | 1 |
| Knowledge | Intercept | 3.72 | 1.39 | 2.68 | .009 |
|  | Group (VP) | -3.29 | 1.95 | -1.69 | 1 |
| Note. *p*-values were adjusted for multiple comparisons using the Holm correction. | | | | | |

| **Table S4** | | | | |
| --- | --- | --- | --- | --- |
| *Post-hoc Pairwise Comparisons for all Main Outcome Measures in the Video Training Group* | | | | |
| Predictor | Estimate | *SE* | *t (df)* | *p* |
| Global skills | 0.14 | 0.06 | 2.56 (83) | .012 |
| Micro skills | -0.02 | 0.12 | -0.15 (83) | .877 |
| Counseling process | 0.16 | 0.08 | 1.84 (83) | .070 |
| Difficult client behavior | 0.28 | 0.08 | 3.55 (83) | <.001 |
| Awareness of values | 0.15 | 0.08 | 2.04 (83) | .044 |
| Clinical self-efficacy | 0.56 | 0.15 | 3.79 (83) | <.001 |
| Overall insecurity | -1.13 | 0.19 | -6.00 (83) | <.001 |
| Building relationship | -0.79 | 0.27 | -2.88 (83) | .005 |
| Complex symptoms | -1.40 | 0.26 | -5.47 (83) | <.001 |
| Practical skills | -1.42 | 0.22 | -6.23 (83) | <.001 |
| Oversights | -1.09 | 0.25 | -4.42 (83) | <.001 |
| Harm/ Suicidality | -0.95 | 0.26 | -3.61 (83) | <.001 |
| Knowledge | 3.73 | 1.40 | 2.66 (83) | .009 |
|  | | | | |

| **Table S5** |  |  |
| --- | --- | --- |
| *Means and Standard Deviations in Perceived Insecurity for the Different Patient Cases in the VP and Video Training Group* | | |
| Group | Patient case | *M* (*SD*) |
| VP training |  |  |
|  | Patient 1 | 3.98 (1.34) |
|  | Patient 2 | 3.74 (1.06) |
|  | Patient 3 | 4.20 (1.24) |
|  | Patient 4 | 4.10 (1.19) |
| Video training |  |  |
|  | Patient 1 | 4.04 (1.03) |
|  | Patient 2 | 4.03 (1.02) |
|  | Patient 3 | 3.95 (1.02) |
|  | Patient 4 | 3.90 (1.03) |
| *Note*. *M* = Mean, *SD* = Standard deviation | | |

| **Table S6** | | |  | |  |  |
| --- | --- | --- | --- | --- | --- | --- |
| *Post-hoc Pairwise Comparisons for Overall Insecurity Across the Different Patient Cases in the VP Training and Video Training Group* | | | | | |  |
| Group | Contrast | *Estimate* | *SE* | *t (df)* | *p* | |
| VP training |  |  |  |  |  | |
|  | Patient 1 – Patient 2 | 0.21 | 0.17 | 1.27 (135) | .652 | |
|  | Patient 1 – Patient 3 | -0.23 | 0.17 | -1.40 (135) | .652 | |
|  | Patient 1 – Patient 4 | -0.14 | 0.17 | -0.82 (135) | .824 | |
|  | Patient 2 – Patient 3 | -0.44 | 0.17 | -2.66 (135) | .053 | |
|  | Patient 2 – Patient 4 | -0.35 | 0.17 | .2.08 (135) | .196 | |
|  | Patient 3 – Patient 4 | 0.10 | 0.17 | 0.58 (135) | .824 | |
| Video training |  |  |  |  |  | |
|  | Patient 1 – Patient 2 | 0.02 | 0.14 | 0.17 (123) | 1 | |
|  | Patient 1 – Patient 3 | 0.10 | 0.14 | 0.69 (123) | 1 | |
|  | Patient 1 – Patient 4 | 0.15 | 0.14 | 1.10 (123) | 1 | |
|  | Patient 2 – Patient 3 | 0.08 | 0.14 | 0.55 (123) | 1 | |
|  | Patient 2 – Patient 4 | 0.13 | 0.14 | 0.95 (123) | 1 | |
|  | Patient 3 – Patient 4 | 0.06 | 0.14 | 0.40 (123) | 1 | |
| *Note*. Patient case 1 served as the reference category for comparisons with each other patient case. *p*-values were adjusted for multiple comparisons using the Holm correction. | | | | | | |

| **Table S7** | |  | |  |  |
| --- | --- | --- | --- | --- | --- |
| *Linear Mixed-Methods Model for Overall Insecurity Across the Different Time Points* | | | | |  |
| Variables | *β* | SE | t | *p* | |
| *VP Training* |  |  |  |  | |
| Intercept | 4.58 | 0.19 | 24.63 | <.001 | |
| Post 1 | -0.34 | 0.17 | -1.94 | 0.054 | |
| Post 2 | -0.63 | 0.17 | -3.65 | <.001 | |
| Post 3 | -0.70 | 0.17 | -4.07 | <.001 | |
| Post 4 | -0.67 | 0.17 | -3.89 | <.001 | |
| *Video Training* |  |  |  |  | |
| Intercept | 4.99 | 0.15 | 32.84 | <.001 | |
| Post 1 | -0.76 | 0.13 | -5.72 | <.001 | |
| Post 2 | -0.92 | 0.13 | -6.94 | <.001 | |
| Post 3 | -1.14 | 0.13 | -8.59 | <.001 | |
| Post 4 | -1.13 | 0.13 | -8.58 | <.001 | |
| *Note.* The pre-training measurement served as the reference category for comparisons with each subsequent post-training time point. | | | | | |

| **Table S8** | |  | |  | | | |
| --- | --- | --- | --- | --- | --- | --- | --- |
| *Linear mixed effects model for psychotherapeutic competence and subscales with patient hours as a moderator* | | | | | | | |
| Predictors |  | | *β* | | SE | t | *p* |
| Global competence |  | |  | |  |  |  |
|  | Intercept | | 3.75 | | 0.12 | 30.79 | < .001 |
|  | Group (VP) | | 0.20 | | 0.17 | 1.16 | 1 |
|  | Time (Post) | | 0.14 | | 0.07 | 2.04 | .088 |
|  | Pat. hours | | 0.00 | | 0.00 | 0.75 | 1 |
|  | Group x Time | | -0.03 | | 0.10 | -0.35 | 1 |
|  | Group x Pat. hours | | -0.00 | | 0.00 | -0.96 | 1 |
|  | Time x Pat. hours | | -0.00 | | 0.00 | -2.04 | .167 |
|  | Group x Time x Pat. hours | | 0.00 | | 0.00 | 1.01 | 1 |
| Micro skills |  | |  | |  |  |  |
|  | Intercept | | 4.08 | | 0.15 | 27.00 | < .001 |
|  | Group (VP) | | 0.12 | | 0.21 | 0.57 | 1 |
|  | Time (Post) | | 0.03 | | 0.16 | 0.21 | 1 |
|  | Pat. hours | | 0.00 | | 0.00 | 0.42 | 1 |
|  | Group x Time | | -0.09 | | 0.22 | -0.40 | 1 |
|  | Group x Pat. hours | | -0.00 | | 0.00 | -0.44 | 1 |
|  | Time x Pat. hours | | -0.00 | | 0.00 | -0.93 | 1 |
|  | Group x Time x Pat. hours | | 0.00 | | 0.00 | 0.51 | 1 |
| Counseling process |  | |  | |  |  |  |
|  | Intercept | | 3.42 | | 0.20 | 16.79 | < .001 |
|  | Group (VP) | | 0.09 | | 0.29 | 0.30 | 1 |
|  | Time (Post) | | 0.27 | | 0.14 | 1.88 | .013 |
|  | Pat. hours | | -0.00 | | 0.00 | -0.36 | 1 |
|  | Group x Time | | 0.18 | | 0.20 | 0.87 | 1 |
|  | Group x Pat. hours | | 0.00 | | 0.00 | 0.34 | 1 |
|  | Time x Pat. hours | | -0.00 | | 0.00 | -0.55 | 1 |
|  | Group x Time x Pat. hours | | -0.00 | | 0.00 | -0.22 | 1 |
| Difficult client behavior |  | |  | |  |  |  |
|  | Intercept | | 3.01 | | 0.16 | 18.70 | < .001 |
|  | Group (VP) | | 0.39 | | 0.23 | 1.70 | 1 |
|  | Time (Post) | | 0.24 | | 0.14 | 1.70 | .312 |
|  | Pat. hours | | 0.00 | | 0.00 | 0.07 | 1 |
|  | Group x Time | | -0.11 | | 0.20 | -0.55 | 1 |
|  | Group x Pat. hours | | -0.00 | | 0.00 | -0.50 | 1 |
|  | Time x Pat. hours | | -0.00 | | 0.00 | -0.77 | 1 |
|  | Group x Time x Pat. hours | | 0.00 | | 0.00 | 0.51 | 1 |
| *Notes*. Pat. hours = Patient hours; Global Competence: Marginal R^2^ = 0.11; Conditional R^2^ = 0.86;  Micro Skills: Marginal R^2^ = 0.03; Conditional R^2^ = 0.48;  Counseling process: Marginal R^2^ = 0.08; Conditional R^2^ = 0.77;  Difficult client behavior: Marginal R^2^ = 0.12; Conditional R^2^ = 0.66;  p-values were adjusted for multiple comparisons using the Holm correction. Given the non-significant results in the linear mixed effect model for *awareness of values,* no moderator analysis for professional experience was conducted. | | | | | | | |

| **Table S9** | |  | |  | | | |
| --- | --- | --- | --- | --- | --- | --- | --- |
| *Linear mixed effects model for self-efficacy with patient hours as moderator* | | | | | | | |
| Predictor |  | | *β* | | SE | t | *p* |
| Clinical self-efficacy | Intercept | | 6.58 | | 0.30 | 21.83 | < .001 |
|  | Group (VP) | | 0.29 | | 0.43 | 0.68 | 1 |
|  | Time (Post) | | 0.83 | | 0.26 | 3.24 | .006 |
|  | Pat. hours | | -0.00 | | 0.00 | -0.28 | 1 |
|  | Group x Time | | -0.24 | | 0.37 | -0.66 | 1 |
|  | Group x Pat. hours | | 0.00 | | 0.00 | 0.43 | 1 |
|  | Time x Pat. hours | | -0.00 | | 0.00 | -0.36 | 1 |
|  | Group x Time x Pat. hours | | -0.00 | | 0.00 | -0.57 | 1 |
| *Notes*. Pat. hours = Patient hours; Marginal R^2^ = 0.13; Conditional R^2^ = 0.68; p-values were adjusted for multiple comparisons using the Holm correction. | | | | | | | |

| **Table S10** | |  |  | | | |
| --- | --- | --- | --- | --- | --- | --- |
| *Linear mixed effects model for insecurity and its subdimensions with patient hours as moderator* | | | | | | |
| Predictors |  | | *β* | SE | t | *p* |
| Overall insecurities | Intercept | | 4.72 | 0.30 | 15.90 | < .001 |
|  | Group (VP) | | -0.60 | 0.42 | -1.43 | 1 |
|  | Time (Post) | | -1.15 | 0.28 | -4.07 | .001 |
|  | Pat. hours | | -0.00 | 0.00 | -0.41 | 1 |
|  | Group x Time | | 0.42 | 0.40 | 1.03 | 1 |
|  | Group x Pat. hours | | -0.00 | 0.00 | -1.5 | 1 |
|  | Time x Pat. hours | | 0.00 | 0.00 | 1.89 | .168 |
|  | Group x Time x Pat. hours | | -0.00 | 0.00 | -0.80 | 1 |
| Building Relationship | | |  |  |  |  |
|  | Intercept | | 3.71 | 0.40 | 9.24 | < .001 |
|  | Group (VP) | | -0.61 | 0.57 | -1.07 | 1 |
|  | Time (Post) | | -0.86 | 0.41 | -2.09 | 1 |
|  | Pat. hours | | -0.00 | 0.00 | -0.59 | 1 |
|  | Group x Time | | 1.19 | 0.59 | 2.03 | .679 |
|  | Group x Pat. hours | | 0.00 | 0.00 | 0.15 | 1 |
|  | Time x Pat. hours | | 0.00 | 0.00 | 1.00 | .826 |
|  | Group x Time x Pat. hours | | -0.00 | 0.00 | -0.27 | 1 |
| Complex Symptoms | | |  |  |  |  |
|  | Intercept | | 5.40 | 0.39 | 13.94 | < .001 |
|  | Group (VP) | | -0.78 | 0.55 | -1.42 | 1 |
|  | Time (Post) | | -1.77 | 0.34 | -5.17 | .000 |
|  | Pat. hours | | -0.00 | 0.00 | -0.06 | 1 |
|  | Group x Time | | 0.26 | 0.49 | 0.54 | 1 |
|  | Group x Pat. hours | | -0.00 | 0.00 | -0.42 | 1 |
|  | Time x Pat. hours | | 0.00 | 0.00 | 1.72 | .171 |
|  | Group x Time x Pat. hours | | -0.00 | 0.00 | -0.53 | 1 |
| Practical skills |  | |  |  |  |  |
|  | Intercept | | 5.21 | 0.34 | 15.34 | <.001 |
|  | Group (VP) | | -0.60 | 0.48 | -1.25 | 1 |
|  | Time (Post) | | -1.48 | 0.43 | -3.41 | .001 |
|  | Pat. hours | | -0.00 | 0.00 | -0.15 | 1 |
|  | Group x Time | | 0.72 | 0.62 | 1.17 | 1 |
|  | Group x Pat. hours | | -0.00 | 0.00 | -0.67 | 1 |
|  | Time x Pat. hours | | 0.00 | 0.00 | 1.06 | 0.816 |
|  | Group x Time x Pat. hours | | -0.00 | 0.00 | -0.28 | 1 |
| Oversights |  | |  |  |  |  |
|  | Intercept | | 5.06 | 0.33 | 15.32 | < .001 |
|  | Group (VP) | | -0.69 | 0.47 | -1.46 | 1 |
|  | Time (Post) | | -1.01 | 0.37 | -2.70 | .045 |
|  | Pat. hours | | -0.00 | 0.00 | -0.72 | 1 |
|  | Group x Time | | 0.49 | 0.53 | 0.91 | 1 |
|  | Group x Pat. hours | | 0.00 | 0.00 | 1.23 | 1 |
|  | Time x Pat. hours | | 0.00 | 0.00 | 1.23 | 1 |
|  | Group x Time x Pat. hours | | -0.00 | 0.00 | -1.58 | 1 |
| Harm/ Suicidality |  | |  |  |  |  |
|  | Intercept | | 4.20 | 0.42 | 9.98 | < .001 |
|  | Group (VP) | | -0.33 | 0.60 | -0.56 | 1 |
|  | Time (Post) | | -0.64 | 0.43 | -1.48 | .036 |
|  | Pat. hours | | -0.00 | 0.00 | -0.15 | 1 |
|  | Group x Time | | -0.58 | 0.61 | -0.96 | 1 |
|  | Group x Pat. hours | | -0.00 | 0.00 | -0.69 | 1 |
|  | Time x Pat. hours | | 0.00 | 0.00 | 1.76 | .106 |
|  | Group x Time x Pat. hours | | -0.00 | 0.00 | -0.30 | 1 |
| *Notes.* Pat. hours = Patient hours; Overall insecurity: Marginal R^2^ = 0.27; Conditional R^2^ = 0.67;  Building relationship: Marginal R^2^ = 0.10; Conditional R^2^ = 0.53;  Complex symptoms: Marginal R^2^ = 0.33; Conditional R^2^ = 0.74;  Practical skills: Marginal R^2^ = 0.28; Conditional R^2^ = 0.41;  Oversights: Marginal R^2^ = 0.20; Conditional R^2^ = 0.41;  Harm/Suicidality: Marginal R^2^ = 0.20; Conditional R^2^ = 0.58;  p-values were adjusted for multiple comparisons using the Holm correction. | | | | | | |

| **Table S11** | |  | |  | | | |
| --- | --- | --- | --- | --- | --- | --- | --- |
| *Linear mixed effects model for psychotherapeutic competence and subscales controlled for initial insecurity* | | | | | | | |
| Predictors |  | | *β* | | SE | t | *p* |
| Global competence |  | |  | |  |  |  |
|  | Intercept | | 5.01 | | 0.19 | 25.86 | < .001 |
|  | Group (VP) | | -0.03 | | 0.09 | -0.37 | 1 |
|  | Time (Post) | | 0.14 | | 0.06 | 2.56 | .013 |
|  | Insecurity | | -0.29 | | 0.04 | -7.98 | .000 |
|  | Group x Time | | 0.00 | | 0.08 | 0.03 | 1 |
| Micro skills |  | |  | |  |  |  |
|  | Intercept | | 5.41 | | 0.26 | 21.17 | < .001 |
|  | Group (VP) | | -0.12 | | 0.13 | -0.87 | 1 |
|  | Time (Post) | | -0.02 | | 0.12 | -0.15 | 1 |
|  | Insecurity | | -0.31 | | 0.05 | -6.46 | .000 |
|  | Group x Time | | 0.10 | | 0.16 | 0.61 | 1 |
| Counseling process |  | |  | |  |  |  |
|  | Intercept | | 5.44 | | 0.28 | 19.54 | < .001 |
|  | Group (VP) | | -0.03 | | 0.13 | -0.25 | 1 |
|  | Time (Post) | | 0.16 | | 0.09 | 1.81 | .017 |
|  | Insecurity | | -0.43 | | 0.05 | -8.22 | .000 |
|  | Group x Time | | 0.12 | | 0.12 | 1.02 | 1 |
| Difficult client behavior |  | |  | |  |  |  |
|  | Intercept | | 4.72 | | 0.25 | 19.24 | < .001 |
|  | Group (VP) | | -0.05 | | 0.12 | -0.42 | 1 |
|  | Time (Post) | | 0.28 | | 0.08 | 3.53 | .000 |
|  | Insecurity | | -0.37 | | 0.05 | -7.99 | .000 |
|  | Group x Time | | -0.04 | | 0.11 | -0.35 | 1 |
| Awareness of values |  | |  | |  |  |  |
|  | Intercept | | 4.46 | | 0.33 | 13.55 | < .001 |
|  | Group (VP) | | 0.06 | | 0.15 | 0.42 | 1 |
|  | Time (Post) | | 0.16 | | 0.08 | 2.02 | 1 |
|  | Insecurity | | -0.06 | | 0.06 | -0.94 | 1 |
|  | Group x Time | | -0.17 | | 0.11 | -1.59 | 1 |
| *Notes*. Global Competence: Marginal R^2^ = 0.39; Conditional R^2^ = 0.77;  Micro Skills: Marginal R^2^ = 0.23; Conditional R^2^ = 0.42;  Counseling process: Marginal R^2^ = 0.40; Conditional R^2^ = 0.74;  Difficult client behavior: Marginal R^2^ = 0.39; Conditional R^2^ = 0.72;  Awareness of values: Marginal R^2^ = 0.02; Conditional R^2^ = 0.73;  p-values were adjusted for multiple comparisons using the Holm correction. | | | | | | | |

| **Table S12** | |  | |  | | | |
| --- | --- | --- | --- | --- | --- | --- | --- |
| *Linear mixed effects model for self-efficacy controlled for initial insecurity* | | | | | | | |
| Predictor |  | | *β* | | SE | t | *p* |
| Clinical self-efficacy | Intercept | | 9.37 | | 0.47 | 20.11 | < .001 |
|  | Group (VP) | | -0.13 | | 0.22 | -0.61 | 1 |
|  | Time (Post) | | 0.56 | | 0.15 | 3.74 | .001 |
|  | Insecurity | | -0.57 | | 0.09 | -6.48 | .000 |
|  | Group x Time | | -0.19 | | 0.21 | -0.91 | 1 |
| *Notes.* Marginal R^2^ = 0.30; Conditional R^2^ = 0.68; p-values were adjusted for multiple comparisons using the Holm correction. | | | | | | | |

| **Table S13** | |  | |  | | | |
| --- | --- | --- | --- | --- | --- | --- | --- |
| *Linear mixed effects model for knowledge controlled for initial insecurity* | | | | | | | |
| Predictor |  | | *β* | | SE | t | *p* |
| Knowledge | Intercept | | 80.49 | | 4.35 | 18.54 | < .001 |
|  | Group (VP) | | -2.56 | | 2.08 | -1.23 | .773 |
|  | Time (Post) | | 3.72 | | 1.39 | 2.68 | 1 |
|  | Insecurity | | -0.59 | | 0.82 | -0.71 | 1 |
|  | Group x Time | | -3.29 | | 1.95 | -1.39 | 1 |
| *Notes.* Marginal R^2^ = 0.30; Conditional R^2^ = 0.68; p-values were adjusted for multiple comparisons using the Holm correction. | | | | | | | |

| **Table S14** | |  |  | | | |
| --- | --- | --- | --- | --- | --- | --- |
| *Linear mixed effects model for insecurity and its subdimensions controlled for initial insecurity* | | | | | | |
| Predictors |  | | *β* | SE | t | *p* |
| Overall insecurities | Intercept | | 1.48 | 0.31 | 4.77 | < .001 |
|  | Group (VP) | | -0.12 | 0.18 | -0.67 | 1 |
|  | Time (Post) | | -1.13 | 0.18 | -6.37 | .000 |
|  | Insecurity | | 0.70 | 0.06 | 12.35 | .001 |
|  | Group x Time | | 0.46 | 0.25 | 1.84 | 1 |
| Building Relationship | | |  |  |  |  |
|  | Intercept | | 1.02 | 0.58 | 1.78 | .079 |
|  | Group (VP) | | -0.22 | 0.31 | -0.73 | 1 |
|  | Time (Post) | | -0.79 | 0.28 | -2.87 | 1 |
|  | Insecurity | | 0.64 | 0.11 | 5.98 | .000 |
|  | Group x Time | | 0.81 | 0.39 | 2.10 | 1 |
| Complex Symptoms | | |  |  |  |  |
|  | Intercept | | 1.49 | 0.47 | 3.20 | .002 |
|  | Group (VP) | | 0.04 | 0.26 | 0.14 | 1 |
|  | Time (Post) | | -1.40 | 0.25 | -5.48 | .000 |
|  | Insecurity | | 0.79 | 0.09 | 9.19 | .000 |
|  | Group x Time | | 0.37 | 0.36 | 1.04 | 1 |
| Practical skills |  | |  |  |  |  |
|  | Intercept | | 2.38 | 0.40 | 5.99 | < .001 |
|  | Group (VP) | | -0.18 | 0.23 | -0.81 | 1 |
|  | Time (Post) | | -1.42 | 0.23 | -6.24 | .000 |
|  | Insecurity | | 0.65 | 0.07 | 8.86 | .000 |
|  | Group x Time | | 0.37 | 0.32 | 1.17 | 1 |
| Oversights |  | |  |  |  |  |
|  | Intercept | | 2.48 | 0.45 | 5.47 | < .001 |
|  | Group (VP) | | -0.17 | 0.25 | -0.66 | 1 |
|  | Time (Post) | | -1.09 | 0.24 | -4.47 | .000 |
|  | Insecurity | | 0.56 | 0.08 | 6.64 | .000 |
|  | Group x Time | | 0.55 | 0.34 | 1.59 | 1 |
| Harm/ Suicidality |  | |  |  |  |  |
|  | Intercept | | 0.02 | 0.53 | 0.04 | .967 |
|  | Group (VP) | | -0.06 | 0.29 | -0.21 | 1 |
|  | Time (Post) | | -0.95 | 0.27 | -3.52 | .001 |
|  | Insecurity | | 0.89 | 0.10 | 9.09 | .000 |
|  | Group x Time | | 0.18 | 0.38 | 0.48 | 1 |
| *Notes.* Overall insecurity: Marginal R^2^ = 0.15; Conditional R^2^ = 0.48;  Building relationship: Marginal R^2^ = 0.22; Conditional R^2^ = 0.37;  Complex symptoms: Marginal R^2^ = 0.44; Conditional R^2^ = 0.46;  Practical skills: Marginal R^2^ = 0.45; Conditional R^2^ = 0.41;  Oversights: Marginal R^2^ = 0.29; Conditional R^2^ = 0.34;  Harm/Suicidality: Marginal R^2^ = 0.40; Conditional R^2^ = 0.47;  p-values were adjusted for multiple comparisons using the Holm correction. | | | | | | |

| **Table S15** | | |  |
| --- | --- | --- | --- |
| *Likelihood-ratio tests comparing the LMMs with feedback to the models without feedback for all outcome variables* | | | |
| Variables | 𝝌2 (df) | *p* | |
| Global competence | 1.65 (1) | .200 | |
| Micro skills | 1.22 (1) | .270 | |
| Counselling process | 0.20 (1) | .653 | |
| Difficult client behavior | 0.19 (1) | .662 | |
| Awareness of values | 6.31 (1) | .012 | |
| Self-efficacy | 3.14 (1) | .077 | |
| Overall insecurities | 1.24 (1) | .265 | |
| Building relationship | 1.14 (1) | .287 | |
| Complex symptoms | 0.49 (1) | .484 | |
| Practical skills | 1.40 (1) | .237 | |
| Oversights | 0.00 (1) | .963 | |
| Harm/Suicidality | 2.48 (1) | .115 | |
| Knowledge | 2.35 (1) | .126 | |
| *Note.* The analysis were conducted with the VP group sample (*n* = 44). | | | |

| **Table S16** | | |  |
| --- | --- | --- | --- |
| *Minimal detectable effects and standardized minimal detectable effects for the interaction of time × group for all outcome variables* | | | |
| **Outcome** | **MES** | **MES_std_** | |
| Global competence | 0.16 | 0.60 | |
| Micro skills | 0.32 | 0.60 | |
| Counselling process | 0.24 | 0.60 | |
| Difficult client behavior | 0.22 | 0.60 | |
| Awareness of values | 0.22 | 0.60 | |
| Self-efficacy | 0.42 | 0.60 | |
| Overall insecurities | 0.53 | 0.60 | |
| Building relationship | 0.77 | 0.60 | |
| Complex symptoms | 0.71 | 0.60 | |
| Practical skills | 0.65 | 0.60 | |
| Oversights | 0.68 | 0.60 | |
| Harm/Suicidality | 0.76 | 0.60 | |
| Knowledge | 3.88 | 0.60 | |
| *Note.* Critical t value and the standard error of the time × group interaction were used to compute the MES values, which were additionally standardized using each model’s residual variance (Lakens (2022)). | | | |

| **Table S17** |  |  |  |  |  |
| --- | --- | --- | --- | --- | --- |
| *Group differences between completers and dropouts* | | | | | |
| Variable |  | *Test statistic*  *t/ χ²* | *df* | *p* | 95% CI |
| Age |  | -1.84 | 54.73 | .071 | [-6.98, 0.30] |
| Practical clinical experience (in years) |  | -1.36 | 67.62 | .178 | [-1.42, 0.27] |
| Patient hours |  | -1.61 | 19.42 | .123 | [-1314.40, 170.06] |
| Gender |  | 4.87 | 2 | .088 | -- |
| Profession |  | 1.40 | 1 | .237 | -- |
| *Notes.* CI = Confidence Interval. Age, practical clinical experience, and patient hours were analyzed using Welch’s t-tests. Gender and profession (categorical variables) were analyzed using chi-square test. | | | | | |

| **Table S18** | |  | |  | | | |
| --- | --- | --- | --- | --- | --- | --- | --- |
| *Linear mixed effects model for psychotherapeutic competence and subscales controlled for study completion duration* | | | | | | | |
| Predictors |  | | *β* | | SE | t | *p* |
| Global competence |  | |  | |  |  |  |
|  | Intercept | | 3.66 | | 0.12 | 31.69 | < .001 |
|  | Group (VP) | | 0.09 | | 0.11 | 0.83 | 1 |
|  | Time (Post) | | 0.14 | | 0.06 | 2.56 | .052 |
|  | Duration | | -0.02 | | 0.02 | -1.30 | 1 |
|  | Group x Time | | 0.00 | | 0.08 | 0.03 | 1 |
| Micro skills |  | |  | |  |  |  |
|  | Intercept | | 3.94 | | 0.15 | 26.77 | < .001 |
|  | Group (VP) | | 0.01 | | 0.15 | 0.09 | 1 |
|  | Time (Post) | | -0.02 | | 0.12 | -0.15 | 1 |
|  | Duration | | -0.01 | | 0.09 | -0.65 | 1 |
|  | Group x Time | | 0.10 | | 0.16 | 0.61 | 1 |
| Counseling process |  | |  | |  |  |  |
|  | Intercept | | 3.44 | | 0.17 | 20.43 | < .001 |
|  | Group (VP) | | 0.16 | | 0.17 | 0.94 | 1 |
|  | Time (Post) | | 0.16 | | 0.09 | 1.81 | 1 |
|  | Duration | | -0.03 | | 0.02 | -1.33 | 1 |
|  | Group x Time | | 0.12 | | 0.12 | 1.02 | 1 |
| Difficult client behavior |  | |  | |  |  |  |
|  | Intercept | | 2.97 | | 0.15 | 20.06 | < .001 |
|  | Group (VP) | | 0.11 | | 0.15 | 0.75 | 1 |
|  | Time (Post) | | 0.28 | | 0.78 | 3.53 | .032 |
|  | Duration | | -0.02 | | 0.02 | -0.86 | 1 |
|  | Group x Time | | -0.04 | | 0.11 | -0.35 | 1 |
| Awareness of values |  | |  | |  |  |  |
|  | Intercept | | 4.28 | | 0.15 | 28.45 | < .001 |
|  | Group (VP) | | 0.10 | | 0.15 | 0.67 | 1 |
|  | Time (Post) | | 0.16 | | 0.08 | 2.02 | 1 |
|  | Duration | | -0.02 | | 0.02 | -1.07 | 1 |
|  | Group x Time | | -0.17 | | 0.11 | -1.59 | 1 |
| *Notes*. Global Competence: Marginal R^2^ = 0.04; Conditional R^2^ = 0.77;  Micro Skills: Marginal R^2^ = 0.00; Conditional R^2^ = 0.42;  Counseling process: Marginal R^2^ = 0.05; Conditional R^2^ = 0.75;  Difficult client behavior: Marginal R^2^ = 0.05; Conditional R^2^ = 0.73;  Awareness of values: Marginal R^2^ = 0.02; Conditional R^2^ = 0.73;  p-values were adjusted for multiple comparisons using the Holm correction. | | | | | | | |

| **Table S19** | |  | |  | | | |
| --- | --- | --- | --- | --- | --- | --- | --- |
| *Linear mixed effects model for self-efficacy controlled for study completion duration* | | | | | | | |
| Predictor |  | | *β* | | SE | t | *p* |
| Clinical self-efficacy | Intercept | | 6.58 | | 0.26 | 25.15 | < .001 |
|  | Group (VP) | | 0.10 | | 0.26 | 0.39 | 1 |
|  | Time (Post) | | 0.56 | | 0.15 | 3.74 | .016 |
|  | Duration | | -0.01 | | 0.03 | -0.31 | 1 |
|  | Group x Time | | -0.19 | | 0.21 | -0.911 | 1 |
| *Notes.* Marginal R^2^ = 0.04; Conditional R^2^ = 0.68; p-values were adjusted for multiple comparisons using the Holm correction. | | | | | | | |

| **Table S20** | |  | |  | | | |
| --- | --- | --- | --- | --- | --- | --- | --- |
| *Linear mixed effects model for knowledge controlled for study completion duration* | | | | | | | |
| Predictor |  | | *β* | | SE | t | *p* |
| Knowledge | Intercept | | 79.65 | | 2.02 | 39.38 | < .001 |
|  | Group (VP) | | -2.16 | | 2.04 | -1.06 | .388 |
|  | Time (Post) | | 3.72 | | 1.39 | 2.68 | 1 |
|  | Duration | | -0.35 | | 0.26 | -1.33 | 1 |
|  | Group x Time | | -3.29 | | 1.95 | -1.69 | 1 |
| *Notes.* Marginal R^2^ = 0.07; Conditional R^2^ = 0.57; p-values were adjusted for multiple comparisons using the Holm correction. | | | | | | | |

| **Table S21** | |  |  | | | |
| --- | --- | --- | --- | --- | --- | --- |
| *Linear mixed effects model for insecurity and its subdimensions controlled for study completion duration* | | | | | | |
| Predictors |  | | *β* | SE | t | *p* |
| Overall insecurities | Intercept | | 4.77 | 0.23 | 20.38 | < .001 |
|  | Group (VP) | | -0.42 | 0.24 | -1.75 | 1 |
|  | Time (Post) | | -1.13 | 0.19 | -5.93 | .000 |
|  | Duration | | 0.04 | 0.03 | 1.35 | 1 |
|  | Group x Time | | 0.46 | 0.27 | 1.71 | 1 |
| Building Relationship | | |  |  |  |  |
|  | Intercept | | 3.97 | 0.33 | 12.19 | < .001 |
|  | Group (VP) | | -0.50 | 0.34 | -1.48 | 1 |
|  | Time (Post) | | -0.79 | 0.28 | -2.87 | 1 |
|  | Duration | | 0.04 | 0.04 | 1.09 | 1 |
|  | Group x Time | | 0.81 | 0.38 | 2.10 | 1 |
| Complex Symptoms | | |  |  |  |  |
|  | Intercept | | 5.44 | 0.31 | 17.57 | < .001 |
|  | Group (VP) | | -0.28 | 0.32 | -0.87 | 1 |
|  | Time (Post) | | -1.40 | 0.25 | -5.48 | < .001 |
|  | Duration | | -0.00 | 0.04 | -0.12 | 1 |
|  | Group x Time | | 0.37 | 0.36 | 1.04 | 1 |
| Practical skills |  | |  |  |  |  |
|  | Intercept | | 5.34 | 0.26 | 20.67 | < .001 |
|  | Group (VP) | | -0.47 | 0.27 | -1.72 | 1 |
|  | Time (Post) | | -1.42 | 0.23 | -6.12 | < .001 |
|  | Duration | | 0.05 | 0.03 | 1.54 | 1 |
|  | Group x Time | | 0.37 | 0.33 | 1.15 | 1 |
| Oversights |  | |  |  |  |  |
|  | Intercept | | 4.93 | 0.27 | 18.55 | < .001 |
|  | Group (VP) | | -0.42 | 0.28 | -1.50 | 1 |
|  | Time (Post) | | -1.09 | 0.25 | -4.47 | .001 |
|  | Duration | | 0.06 | 0.03 | 1.88 | 1 |
|  | Group x Time | | 0.55 | 0.34 | 1.59 | 1 |
| Harm/ Suicidality |  | |  |  |  |  |
|  | Intercept | | 4.17 | 0.34 | 12.11 | < .001 |
|  | Group (VP) | | -0.44 | 0.35 | -1.25 | 1 |
|  | Time (Post) | | -0.95 | 0.27 | -3.52 | .003 |
|  | Duration | | 0.05 | 0.04 | 1.13 | 1 |
|  | Group x Time | | 0.18 | 0.38 | 0.48 | 1 |
| *Notes.* Overall insecurity: Marginal R^2^ = 0.16; Conditional R^2^ = 0.48;  Building relationship: Marginal R^2^ = 0.04; Conditional R^2^ = 0.37;  Complex symptoms: Marginal R^2^ = 0.14; Conditional R^2^ = 0.47;  Practical skills: Marginal R^2^ = 0.43; Conditional R^2^ = 0.21;  Oversights: Marginal R^2^ = 0.12; Conditional R^2^ = 0.34;  Harm/Suicidality: Marginal R^2^ = 0.08; Conditional R^2^ = 0.47;  p-values were adjusted for multiple comparisons using the Holm correction. | | | | | | |
